# Human Biomonitoring of Mycotoxins in Blood, Plasma and Serum in Recent Years: A Review

**DOI:** 10.3390/toxins12030147

**Published:** 2020-02-27

**Authors:** Beatriz Arce-López, Elena Lizarraga, Ariane Vettorazzi, Elena González-Peñas

**Affiliations:** 1 Department of Pharmaceutical Technology and Chemistry; Universidad de Navarra, 31008 Pamplona, Navarra, Spain; barce@alumni.unav.es (B.A.-L.); mgpenas@unav.es (E.G.-P.); 2 Department of Pharmacology and Toxicology. School of Pharmacy and Nutrition, 31008 Pamplona, Navarra, Spain; avettora@unav.es; 3 IdiSNA, Institute for Health Research, 31008 Pamplona, Navarra, Spain

**Keywords:** blood, HBM, mycotoxins, plasma, serum

## Abstract

This manuscript reviews the state-of-the-art regarding human biological monitoring (HBM) of mycotoxins in plasma, serum and blood samples. After a comprehensive and systematic literature review, with a focus on the last five years, several aspects were analyzed and summarized: (a) the biomarkers analyzed and their encountered levels, (b) the analytical methodologies developed and (c) the relationship between biomarker levels and some illnesses. In the literature reviewed, aflatoxin B1-lysine (AFB1-lys) and ochratoxin A (OTA) in plasma and serum were the most widely studied mycotoxin biomarkers for HBM. Regarding analytical methodologies, a clear increase in the development of methods for the simultaneous determination of multiple mycotoxins has been observed. For this purpose, the use of liquid chromatography (LC) methodologies, especially when coupled with tandem mass spectrometry (MS/MS) or high resolution mass spectrometry (HRMS) has grown. A high percentage of the samples analyzed for OTA or aflatoxin B1 (mostly as AFB1-lys) in the reviewed papers were positive, demonstrating human exposure to mycotoxins. This review confirms the importance of mycotoxin human biomonitoring and highlights the important challenges that should be faced, such as the inclusion of other mycotoxins in HBM programs, the need to increase knowledge of mycotoxin metabolism and toxicokinetics, and the need for reference materials and new methodologies for treating samples. In addition, guidelines are required for analytical method validation, as well as equations to establish the relationship between human fluid levels and mycotoxin intake.

## 1. Introduction

Mycotoxins are fungal secondary metabolites produced by phytopathogenic fungi such as *Aspergillus*, *Penicillium*, *Fusarium*, and *Alternaria* toxigenic species [1].

The designation of mycotoxins includes a group of highly heterogeneous compounds, in terms of chemical structure and toxicological properties [2], with a low molecular mass [3]. The classification of mycotoxins is a complex task since they have diverse chemical structures and biosynthetic origins and, also, they are produced by a great variety of fungal species. Moreover, it should be noted that the same mycotoxin can be produced by several fungal species; for example, ochratoxin A (OTA) can be produced by *Penicillium verrucosum*, *Aspergillus ochraceus* and *Aspergillus carbonarius*. In addition, the same fungal species can produce more than one mycotoxin. This is the case for *Fusarium graminearum,* which produces zearalenone (ZEA) and deoxynivalenol (DON) [3]. Aflatoxins (AFs) and ochratoxins (produced by *Aspergillus spp*. and *Penicillium spp*.), fumonisins (FBs), trichothecenes and ZEA (produced by *Fusarium spp*.) and patulin (PAT) and citrinin (CIT) (produced by *Penicillium spp*.) are the most commonly observed mycotoxins that pose serious health threats to humans and animals [4].

Fungi, and their metabolites, contaminate raw materials that are usually used in the preparation of human food and animal feed. The main crops affected are grains (rice, wheat, rye, barley, corn, soybeans…), dried fruits, nuts, coffee and spices. Contamination may occur naturally during the growth of the crop or may be a result of improper transport and storage processes. The presence of these metabolites is also known to be largely dependent on environmental factors such as temperature and humidity [5], and thus is dependent on climate [6,7].

Fungal contamination control measures, some prevention strategies and an improved processing technologies can help limit mycotoxin contamination [8]. However, and despite these efforts, up to 60%–80% of food crops are still contaminated by mycotoxins [9]. Indeed, cereal-based foods, beverages and products of animal origin commonly present with mycotoxins [4,10,11]. Moreover, food processing does not completely remove these toxic compounds [8], which remain stable in human gastric acid at low pH [12]. For all these reasons, their presence in human food and animal feed represents a matter of great concern [9,13], due not only to the negative effect on human and animal health, but also to the deep impact that losses of contaminated crops have on the global economy [14].

Human exposure to mycotoxins takes place through the consumption of contaminated food such as cereals. It can also occur through the ingestion of products of animal origin, such as eggs and milk, if the animals have previously been fed with contaminated feed [14,15,16]. Additionally, humans can be exposed to mycotoxins by inhalation and dermal contact with contaminated dust or mold [17].

Mycotoxins cause toxic responses known as mycotoxicoses [18]. Carcinogenicity, hepatotoxicity, nephrotoxicity and endocrine disorders have been related to chronic exposure to low levels of mycotoxins [19]. In addition, mycotoxins may produce metabolic and biochemical deficiencies, allergic reactions, immune diseases, reproductive deficiencies, fetal alterations and death [20,21]. The impact of mycotoxins on human health depends on the type of toxin, its metabolism, pharmacokinetics and the accumulation of the mycotoxin, exposure conditions and the age, gender, immune system and health status of the exposed individual [22].

For all of the above reasons, understanding and controlling human and animal exposure to mycotoxins is a key concern [22].

There are two approaches to evaluating human exposure to mycotoxins. The first involves analyzing the occurrence of toxins in food commodities and then combining these data with information on food consumption (external exposure). The second involves biomonitoring a biomarker in any human fluid or tissue (e.g., blood, urine) (internal exposure) [23,24]. The assessment of human exposure to mycotoxins has traditionally been performed by means of the former methodology [25,26]. In this case, the free or parent form of the mycotoxin is usually determined. For this purpose, several analytical methods have been developed based on either chromatography or immunochemistry, such as the Enzyme-Linked Immunosorbent Assay (ELISA). In fact, numerous articles are available on the occurrence of mycotoxins in many food products and their derivatives [4,27].

However, this approach presents several disadvantages. The first stems from the evaluation of mycotoxin levels in food, since their distribution is not homogeneous. In addition, some mycotoxins may be linked to matrix substances or may be biologically or chemically modified in the raw material [28], and are therefore not detected during the analytical procedure. This results in an underestimated exposure level [26]. Moreover, it is difficult to obtain accurate data on food consumption. Finally, the presence of these toxins in food does not necessarily imply that humans have been exposed to them. Their bioavailability can vary depending on several factors, such as the composition of the food, the treatment the food has undergone [8] and inter-individual differences. It is therefore difficult to carry out accurate risk assessments based on these data alone.

In this context, human biological monitoring (HBM)-that is, the analysis of mycotoxin biomarkers in body fluids and tissues [29] (internal exposure)-represents a more effective strategy for investigating human exposure [30,31] than the evaluation of food contamination [32], although the two approaches are complementary to each other. The advantage of estimating exposure through mycotoxin levels in biological matrices is that identification of the contamination source involved (ingestion of contaminated food or inhalation of contaminated air) is not necessary. This method requires a single determination per person and bypasses the problems associated with food sampling methods and consumption data collection. Biomonitoring should therefore be performed continuously worldwide to control mycotoxin exposure in humans [22].

A number of reviews have already been carried out on the topic of HBM of mycotoxins. Most of these reviews focused on OTA. Fromme et al. (2012) [33], Malir et al. (2016) [34], Soto et al. (2015) [35] and Ropejko et al. (2019) [36] reviewed the OTA levels in human biological fluid samples. Soto et al. (2015) [35] concluded that OTA levels in body fluids are good biomarkers of human exposure to this mycotoxin. Also, Coronel et al. (2010) [24] summarized the OTA plasma levels in different countries up to 2008. In this review, it was noted that OTA occurred in 74% of the analyzed samples. The authors also reviewed the factors underpinning OTA presence in plasma and indicated that statistical differences were reported among individuals based on factors such as age and gender, and especially season and geographical location. This latter conclusion was also reached by Ropejko et al. (2019) [36]. The authors recommended that further studies on OTA human exposure should be carried out, particularly in special groups such as children, older people and individuals following special diets [24].

In 2012, Leong et al. [37] published a short review about the presence of AFs in human fluids. Escrivá et al. (2017) [31] reviewed the presence of mycotoxins in biological samples from several species. The matrices reviewed in humans were urine, serum, feces and breast milk. With respect to human serum samples, in studies reviewed up to 2016, OTA was the most widely studied mycotoxin (11/14 studies). Aflatoxin B1 (AFB1), ochratoxin α (OTα), CIT, enniatins (Ens) and beauvericin (BEA) were also analyzed in some papers referenced by the authors, but to a lesser extent. Waseem et al. (2014) [17] summed up the presence of mycotoxins in different human biological matrices and, in terms of human blood and serum, included papers up to 2013. The most recently published review, by Al-Jaal et al. (2019) [22] summarized the presence of AFs, FBs, OTA, ZEA and DON in biological fluids, especially in urine; however, regarding plasma/serum samples, most of the articles reviewed were published before 2015. Marin et al. (2018) [38] reviewed the methodology for detecting mycotoxin biomarkers in human samples. Finally, Tesfamariam et al. (2019) [39] summarized the evidence of the relationship between exposure to AFs and FBs and some diseases in children.

However, the number of recently published papers on this topic has grown; most of them evaluated the determination and occurrence of multiple mycotoxins in human plasma or serum. For this reason, the aims of this updated, comprehensive and systematic review were to address the progress made in this area over the last five years, to summarize the information regarding the possible association between exposure to certain diseases and the mycotoxin levels encountered in these matrices; and to outline some of the challenges associated with the development of mycotoxin HBM.

## 2. Results

In this section, data retrieved from the articles selected after a systematic review are presented. Altogether, 164 articles were selected. The strategy of the revision is indicated in the Material and Methods section.

### 2.1. Human Biomonitoring of Mycotoxin Exposure

HBM is increasingly being accepted as an efficient way of assessing human exposure, through any route, to food contaminants such as mycotoxins without the need to identify the main source of exposure [40,41,42]. It is based on the accurate measurement of biomarkers in human fluids and tissues [43]. Human biomonitoring requires validated biomarkers, validated analytical methods and easily accessible biological matrices such as urine, plasma, serum and breast milk, among others.

Biomarkers were defined by Vidal et al. (2018) as characteristics that are objectively measured and evaluated as an indicator of normal biological or pathogenic processes, pharmacologic responses to a therapeutic intervention or toxic responses to a toxic agent [43]. The three accepted categories of biomarkers are: of exposure, of response (or toxic effect) and of susceptibility [40,43].

Mycotoxin biomarkers have been defined as the compounds (e.g., parent toxins and/or a metabolite) or the products of their interaction with target molecules (e.g., protein or DNA adducts and glucuronide conjugates) that can be measured in body fluids or tissues and can be correlated with ingested mycotoxins [38]. Duarte et al. (2011) [40] suggested that a good biomarker should be quantitative, sensitive, non-invasive, specific, and easily measurable, and that it should relate to the biochemical mechanism and work at realistic doses. However, it must be noted that the correlation between any biomarker in a body fluid or tissue and exposure depends on the type of matrix sampled, the time between exposure and sampling, the pharmacokinetics of the mycotoxin and the detection capacity of the analytical method used to quantify the biomarker [29]. The appropriate selection of representative biomarkers to be analyzed for each mycotoxin is crucial. This is why further studies on the metabolism of mycotoxins in humans should be performed. A good example is Al-Jaal et al. (2019) [22], who reviewed the metabolism of some mycotoxins in the human body and the biomarkers that can be used to assess mycotoxin exposure. This knowledge will be crucial for determining human exposure to mycotoxins through analysis of biological fluids or tissues.

**Figure 1:**
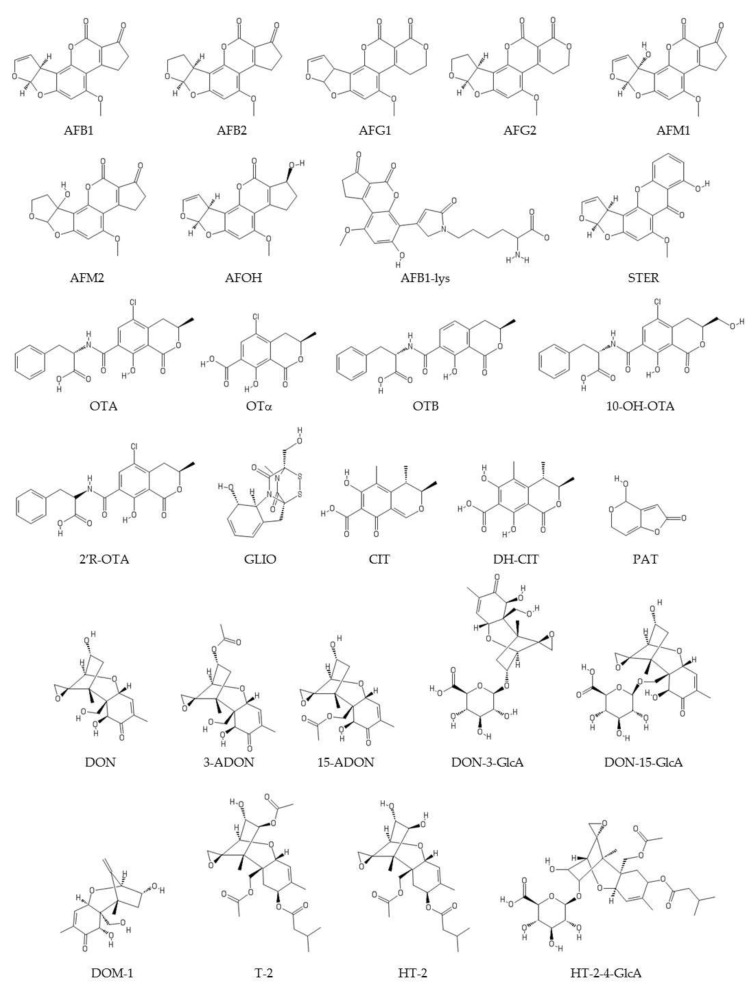
Structures of the studied analytes in the retrieved articles. AFB1: aflatoxin B1; AFB2: aflatoxin B2; AFG1: aflatoxin G1; AFG2: aflatoxin G2; AFM1: aflatoxin M1; AFM2: aflatoxin M2; AFOH: aflatoxicol; AFB1-lys: adduct of AFB1 with lysine; STER: sterigmatocystin; OTA: ochratoxin A; OTα: ochratoxin α; OTB: ochratoxin B; 10-OH-OTA: 10-hydroxyochratoxin A; 2´R-OTA: 2’R-ochratoxin A; GLIO: gliotoxin; CIT: citrinin; DH-CIT: dihydrocitrinone; PAT: patulin; DON: deoxynivalenol; 3-ADON: 3-acetyldeoxynivalenol; 15-ADON: 15-acetyldeoxynivalenol; DON-3-GlcA: deoxynivalenol-3-glucuronide; DON-15-GlcA: deoxynivalenol-15-glucuronide; DOM-1: deepoxy-deoxynivalenol; T-2: T-2 toxin; HT-2: HT-2 toxin; HT-2-4-GlcA: HT-2-toxin-4-glucuronide. Modified from PubChem (https://pubchem.ncbi.nlm.nih.gov).

**Figure 2:**
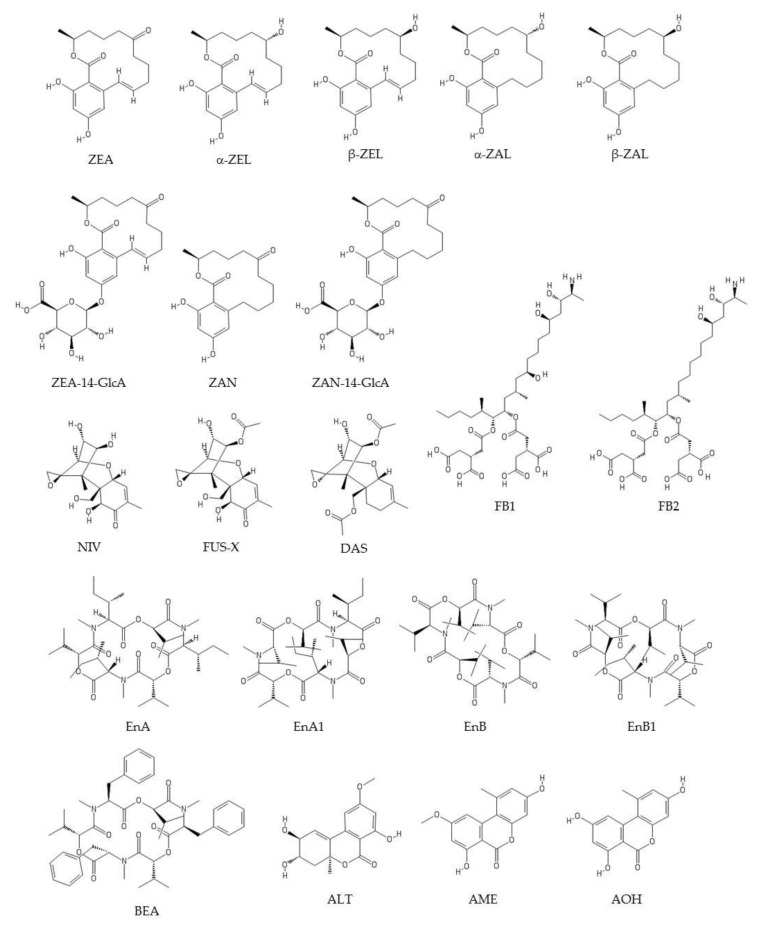
Structures of the studied analytes in the retrieved articles. ZEA: zearalenone; α-ZEL: α-zearalenol; β-ZEL: β-zearalenol; α-ZAL: α-zearalanol; β-ZAL: β-zearalanol; ZEA-14-GlcA: zearalenone-14- glucuronide; ZAN: zearalanone; ZAN-14-GlcA: zearalanone-14- glucuronide; FB1: fumonisin B1; FB2: fumonisin B2; NIV: nivalenol; FUS-X: fusarenon-X; DAS: diacetoxyscirpenol; EnA: enniatin A; EnA1: enniatin A1; EnB: enniatin B; EnB1: enniatin B1; BEA: beauvericin; ALT: altenuene; AME: alternariol monomethyl ether; AOH: alternariol. Modified from PubChem (https://pubchem.ncbi.nlm.nih.gov).

Structurally, mycotoxins can occur in three possible forms [28,44,45]. “Unmodified” forms are biosynthesized by fungal metabolism (e.g., OTA, AFB1, ZEA, DON, fumonisin B1 (FB1), PAT) and refer to the basic or free forms of mycotoxin structures. “Matrix-associated” mycotoxins form complexes with matrix compounds. Examples include FBs bound to proteins and OTA bound to polysaccharides. Finally, “modified” mycotoxins have undergone chemical or biological modification to their structure. These modified mycotoxins can be produced by fungi, such as, for instance, ZEA-14-sulfate. Also, plants and animals are able to modify toxins as a result of their metabolic processes; for instance, plants can produce DON 3-glucoside and ZEA-14-glucoside, and animals DON-3/8/15-glucuronides and HT2-3/4-glucuronides. These changes to the structure of mycotoxins occur during metabolic reactions. Other modifications are possible, such as the formation of deepoxy-deoxynivalenol (DOM-1) by animal and human microbiota. Modified mycotoxins may also form during food processing and, in some cases, can be reconverted to the parent toxin during animal or human metabolism [44]. Exposure to modified mycotoxins therefore poses an additional risk to human health. Modified mycotoxins can become free toxins in the digestive system, thus increasing exposure to these toxins [46,47]. They may be just as toxic as the parent compound (e.g., if they follow the same metabolic process), less toxic (if the modified form has not been transformed, or has been only partially transformed), or even more toxic [46]. For these reasons, all forms of mycotoxin should be included in HBM (e.g., free forms, metabolites, conjugates, etc.) [22]. The structures and some chemical characteristics of the studied analytes in the retrieved articles are shown in Figure 1 and Figure 2 and Table 1.

Until now, only a few biomarkers have been validated for estimating mycotoxin exposure, namely, DON-glucuronides in urine, adduct of AFB1 with albumin (AFB1-alb) and AFB1-lys in human plasma, and AFB1-N7-guanine in urine [43]. Moreover, there is a shortage of HBM studies and biomarker definitions with respect to some mycotoxins, e.g., T-2 toxin (T-2), HT-2 toxin (HT-2), sterigmatocystin (STER) and nivalenol (NIV).

On the other hand, HBM requires sufficiently sensitive and validated analytical methods. There are three approaches to determining biomarkers of mycotoxins in biological matrices: direct, indirect and non-targeted analysis. Direct analysis uses standardized analytical methods that have been properly optimized and validated. However, this approach can be applied to parent compounds only, because just a few modified mycotoxins are available as reference substances. To overcome this problem, indirect determination can be used. In this case, modified mycotoxins are transformed into their free forms, which can then be analyzed using routine methods. Hydrolysis, reduction and other specific reactions can be used. Until now, direct and indirect methods based on liquid chromatography tandem mass spectrometry (LC-MS/MS) have been chosen to identify and quantify unmodified and modified mycotoxins [28,45].

While the potential of tandem mass spectrometry for the quantitative determination of multiple mycotoxins has been widely documented, there is currently growing interest in evaluating new mass spectrometer (MS) detection approaches, based mainly on high-resolution MS (HRMS), for a more complex task, i.e., the determination of unknown mycotoxin derivatives. In this case, non-targeted analysis is required and LC-HRMS is usually employed [43]. As an example, MS techniques based on high-resolution Orbitrap MS have advanced to the point that they now include the non-targeted analysis of fungal metabolites [48,49].

Furthermore, HBM requires easily accessible biological matrices such as urine, serum, plasma and breast milk. Urine analysis presents some advantages because sampling is non-invasive and collection is easy and it contains biomarkers of almost all mycotoxins; therefore, it is very often the matrix of choice for estimating human exposure [50,51]. Nevertheless, urine biomarkers reflect day-to-day variations in mycotoxin intake, so samples must be taken at different times over a 24-h period. In addition, there is variability in the volume of collected urine at different times and among individuals; this variability leads to changes in the concentration of excreted compounds in the samples. Several methods can be applied in order to reduce this variability [52]. Among them, the normalization of the mycotoxin levels for creatinine concentration (µg mycotoxin L^-1^ urine/g creatinine L^−1^ urine) is one of the most employed [30,53]. However, this procedure also has some drawbacks, since it is not clear if the mycotoxin/creatinine ratio can be used for interindividual comparison. Creatinine secretion can vary among different people related to muscle mass, sex, age, season, diet, etc. [52]. Clearly, breast milk can be used to monitor only lactating women; nonetheless, it is an excellent information source for exposure in breastfed babies. Serum and plasma matrices at least have the advantage of requiring less sensitive methods because they contain higher levels of compounds [48]. In addition, while urinary excretion normally indicates recent mycotoxin intake, plasma and serum measurements indicate long-term exposure [40]. However, they are limited in that they require invasive collection methods and medical professionals.

Another important aspect is that the level of a biomarker in the same individual usually varies over time. Coronel et al. (2010) [24] reviewed this aspect in relation to OTA and suggested that determining this mycotoxin in plasma would be useful if it were used to characterize populations instead of individuals. Toxicokinetic studies of different biomarkers in humans would be highly beneficial, but they are greatly limited for obvious ethical reasons.

### 2.2. Mycotoxin Determination in Human Blood, Plasma and Serum

**Figure 3:**
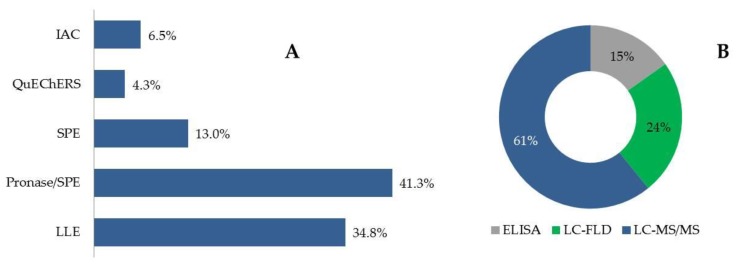
Extraction (**A**) and detection techniques (**B**) for mycotoxin determination in human blood/plasma/serum according to the articles reviewed on these matrices. The percentage of articles using each technique is indicated. IAC: immunoaffinity columns; LLE: liquid–liquid extraction; QuEChERS: Quick Easy Cheap Effective Rugged and Safe; SPE: solid-phase extraction.

Due to the complexity of blood, plasma and serum samples, matrix components might interfere in analyte retention, in addition to reducing purification, recovery and method sensitivity and producing matrix effects when MS detectors are used. For these reasons, all the papers reviewed included the different steps designed to extract analytes and cleanup matrix components. The different methodologies used for preparing samples and determining mycotoxins in plasma, serum and blood samples over the last five years are shown in Table 2 and Figure 3.

The most widely used mycotoxin-related extraction, cleanup and enrichment procedure was solid-phase extraction (SPE), based on the retention of analytes on a fixed support in a cartridge. SPE was used in 54.3% of the reviewed papers; most of them (76%) used it as a purification method in samples previously digested with a mixture of proteinases (Pronase^®^) to detect adducts of aflatoxin (AF-adducts). SPE can be used to purify and preconcentrate analytes. Liquid–liquid extraction (LLE) with different organic solvents was also widely used. Acetonitrile (ACN) [23,54,55,56], chloroform [57], chloroform/isopropanol [55], ethylacetate (EtOAc) [48,58], diethyl ether [58] and mixtures of these with water, such as H_2_O/acetone/ACN [59,60,61,62,63], have proven to be the most efficient solvents for mycotoxin LLE extraction. An acid solution can help the extraction process by disrupting interactions between the toxins and sample constituents, such as proteins. Thus, ACN with formic or acetic acids [64,65] has also been used to extract mycotoxins. Therefore, the main sample preparation procedures described in the reviewed literature from the last five years are consistent with those included in the review by Escrivá et al. (2017) [31], although SPE took over from LLE as the most common technique. The use of Captiva^®^ EMR-Lipid cartridges has been described as a novel procedure in order to reduce matrix effects by eliminating phospholipids from plasma during sample preparation [66]. Apart from this one, no innovative improvements to the sample preparation process have been reported.

Quick, easy, cheap, effective, rugged, and safe (QuEChERS) (based on extraction with ACN), and immunoaffinity columns (IAC) (a variation of SPE in which the fixed support contains specific antibodies for mycotoxin retention) were not widely used (Figure 3). The QuEChERS procedure is based on the extraction of analytes from an aqueous matrix using a polar organic solvent (MeOH or ACN). ACN and MeOH are miscible with water; therefore, the addition of salts to the aqueous phase is needed in order to achieve the separation of the two aqueous-organic phases. Also, salt addition favors the distribution of mycotoxins to the organic solvent. However, when very polar mycotoxins (such as PAT or FBs) are in the samples, high levels of organic solvent during the extraction procedure (that contributes to less polar mycotoxin extraction) lead to low recovery values, probably because they remain in the discarded aqueous phase during the extraction [67]. In these cases, a high percentage of water is needed [53] and less polar mycotoxins can be non-extracted. In the case of IAC, this was due to its specificity. This characteristic represents its main advantage for its use in single analysis; nevertheless, it is a drawback in multi-mycotoxin analysis, because it prevents the simultaneous retention of several compounds and their related metabolites.

In addition, some authors have added an enzymatic de-conjugation method to evaluate phase II metabolites. Plasma or serum samples were incubated with β-glucuronidase, or β-glucuronidase/sulfatase at 37 °C overnight, then the selected extraction procedure was applied [55,64,68,69].

With respect to the detection and quantification of mycotoxin in blood, plasma and serum samples, several techniques were applied (Figure 3). Most of the papers referenced focused on determining one mycotoxin or some structurally related mycotoxins (78% of the studies reviewed). In other cases, the objective was to determine multiple mycotoxins simultaneously.

ELISA is a routine screening tool for the rapid monitoring of mycotoxins that offers high sensitivity, affordability and ease of analysis [70]. A wide range of commercial ELISA-based kits is available for different matrices, including human plasma, which makes it a highly versatile methodology. However, the potential for cross-reactivity with metabolites of target compounds or matrix components can give rise to overestimated values [71]. For this reason, AOAC International has not approved any ELISA method [72] and positive results must be confirmed (e.g., through chromatographic methods). Another shortcoming of ELISA is that it relies on specific antibodies for each mycotoxin; therefore, it is not appropriate for multi-mycotoxin determination. In fact, ELISA was applied in just 15% of all studies reviewed [73,74,75,76,77,78,79].

Liquid chromatography (LC), performed on reversed-phase columns, has become the main tool for determining mycotoxins in human fluids, including human blood, plasma and serum, and was used in 86% of all publications referenced. Advances in high-sensitivity detectors, in LC pump design and in column-packing materials have led to better limits of detection and improved chromatographic performance [80]. The introduction of Ultra-LC (UHPLC), characterized by uniform column packing material with a particle size of less than 2–3 µm and new pumps and detectors, has represented an improvement, given that it achieves shorter run times, reduces solvent consumption and improves chromatographic resolution and efficiency. UHPLC was reported in 42.8% of the chromatographic methods summarized in this review [48,53,58,64,65,68,69,81,82,83].

The fluorescence detector (FLD) provides high sensitivity and selectivity, is easy to use, is less expensive than other methods (e.g., MS), and presents an advantage over MS, i.e., there is no matrix effect [84]. However, sample preparation for this technique is time-consuming [70]. Liquid chromatography coupled with fluorescence detection (LC-FLD) was used frequently (24% of the methods reviewed) [54,55,57,58,81,85,86,87,88,89,90,91,92].

**Figure 4:**
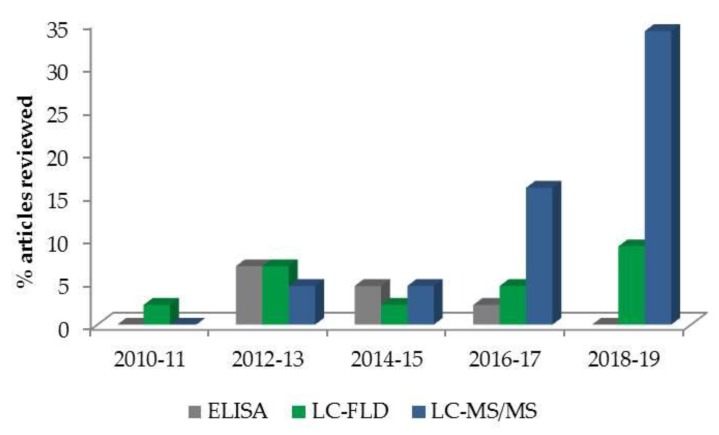
Use of different analytical techniques for mycotoxin determination in human blood/plasma/serum across the years (in relation to the articles reviewed). The percentage of articles using each technique is shown.

The use of MS detectors in mycotoxin determination provides a valuable and confirmatory technique. The literature on mycotoxin determination in biological fluids is scarce, but chromatographic systems coupled with tandem MS are a reference technique for this purpose and were used in 58% of all articles referenced. Although the matrix effect on the MS signal is significant, instrumentation is expensive, and it requires high technical personnel training. MS detectors have key advantages, including high selectivity and sensitivity and the possibility of structural elucidation. Although several applications of LC-MS and LC-MS/MS have been developed for single-mycotoxin analysis, this methodology offers the possibility of simultaneously detecting multiple mycotoxins in a single run. Therefore, an increasing number of articles focused on biomonitoring several mycotoxins in human plasma, serum and blood samples, with a tendency towards LC-MS/MS (Figure 4). The main ionization sources employed were electrospray ionization (ESI) and atmospheric pressure chemical ionization (APCI). Regarding the mass analyzers, the triple quadrupole (QqQ) and the quadrupole-ion trap (QTrap) were the most commonly used (57% and 32%, respectively).

Recent advances in high-resolution mass spectrometry (HRMS) [48,49,81] and the use of multi-mycotoxin biomarker databases have improved the identification and validation of biomarkers of exposure and revealed new mycotoxin metabolites [43]. These studies have contributed to a more in-depth knowledge of the biomarkers of exposure in humans.

Other approaches have also been investigated during the last few years for developing new methodologies, based on biosensors, in order to determine mycotoxins in plasma, serum or blood. A biosensor is “any measuring device containing a biologically derived sensing (so-called biorecognition) element intimately associated with a sensor element (physicochemical transducer)” [93]. These devices have the advantage of being portable, sensitive and not as expensive as traditional approaches [93]. Recently, excellent reviews have been published regarding the development and the applicability of biosensors for mycotoxin determination [94,95,96,97,98]. Very few biosensor methodologies focusing on the analysis of mycotoxins in biological fluids have been published in the last 5 years. Moreover, all of them are limited to single-mycotoxin determination. Abnous et al. (2017) [99] obtained a good linear relationship in the range of 7–500 pg/mL, achieving a limit of detection (LOD) of 2.8 pg/mL and recovery values from 95.4% to 108.1% when serum samples were spiked with AFB1. Also, these authors demonstrated that their methodology was selective as regards to other possible interfering mycotoxins, such as AFM1, OTA, ZEN, AFB2 and DON. Beheshti-Marnani et al. (2019) [100] obtained a LOD of 0.07 nM and recovery values from 97.64% to 104.0% in AFB1 spiked blood samples. In the case of OTA, Nameghi et al. (2016) [101] developed a fluorescent aptasensor intended to be used in serum samples, obtaining a LOD of 74.3 pg/mL. Wang et al. (2016)[102] developed a methodology based on biosensors for the determination of OTA in human serum in which the samples do not need pretreatment, only dilution, before analysis. The recovery rate was from 92% to 101.9% in spiked serum samples. For ZEA, some biosensors have also been investigated. Jiang et al. (2019) [103] determined ZEA levels in plasma and urine samples collected from healthy volunteers using electrochemical immunosensors. ACN acidified with 1% formic acid was added to the samples to precipitate proteins. After vortexing and centrifuging, the supernatant was dried (N_2_) at 30 °C. Finally, the residue was dissolved in phosphate buffer solution (PBS) at pH 6.5 before the analysis. The recovery rates were from 90.2% to 107.4% for plasma samples and 90.3% to 106.3% for urine samples, and a LOD of 0.005 ng/mL was indicated for both matrices. This work was the only one that achieved the determination of the mycotoxin in real samples (two positive plasma samples and two positive urine samples). In addition, the obtained results were compared with those obtained using UHPLC-MS/MS and a good correlation for the mycotoxin levels by the two methodologies was found. Nevertheless, and despite the advantages of biosensors and their foreseeable interest for mycotoxin determination in the future, nowadays, biosensors are still under development and research [97,98] and new advances should be made in order to enhance their specificity and sensitivity [98].

There are some difficulties in the development and validation of analytical methods for HBM. First, standards of analytes are needed to carry out the method validation. They do not exist for some mycotoxins and obtaining them for the majority of metabolites is a complex task. Moreover, they are very expensive when available for purchase. In addition, when MS/MS detectors are used, it is often difficult to obtain matrix-free samples for the preparation of calibrators; in these cases, the use of labeled compounds can help. However, these labeled compounds are expensive and, more importantly, can have different retention times, recoveries and matrix effects than those of the parent compounds, and thus these factors should be taken into account during method validation [104,105]. Also, matrix reference materials with a known concentration of biomarkers are not at researchers’ disposal for method validation.

Though validation is mandatory [106], there are no guidelines for the validation of mycotoxin quantification methods in human body fluids. Some authors [53,64,65] referred to EU Commission Decision No. 2002/657/EC [107], which mentions the rules for the analytical methods to be used in the testing of official samples (residues in live animals and animal products), or the EU Commission Regulation (EC) No. 401/2006 [108] which refers to methods for analyzing mycotoxins in foodstuffs. Other authors [48,64] validated their methodologies in accordance with guidelines on bioanalytical method validation issued by the Food and Drug Administration (FDA) [109] or the European Medicines Agency [110] which refer to drug development. Validation requires aspects such as criteria for determining the different validation parameters and controlling the analysis, along with the required limits of detection or quantification values for mycotoxin biomarkers in biological fluids.

### 2.3. Mycotoxin Biomarkers in Human Blood, Plasma and Serum

**Figure 5:**
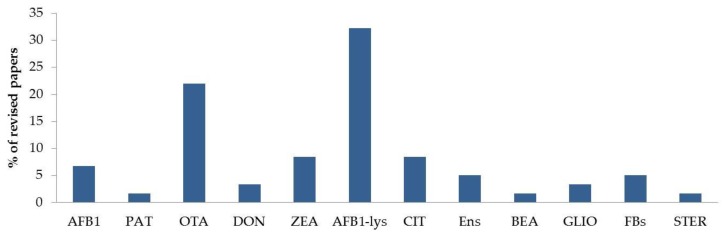
Biomarkers of mycotoxins detected in human plasma or serum samples (according to the articles reviewed). The percentage of the retrieved papers that analyze each of the biomarkers is shown. AFB1: aflatoxin B1; AFB1-lys: adduct of AFB1; BEA: beauvericin; CIT: citrinin; DON: deoxynivalenol; Ens: enniatins; FBs: fumonisins; GLIO: gliotoxin; OTA: ochratoxin A; PAT: patulin; STER: sterigmatocystin; ZEA: zearalenone.

In the last five years, mycotoxin HBM in blood, plasma and serum has been carried out primarily through the analysis of parent compounds, e.g., OTA, ZEA, AFB1, aflatoxin B2 (AFB2), aflatoxin G1 (AFG1) and aflatoxin G2 (AFG2). In some cases, HBM was carried out by determining adducts. For instance, the measurement of AFB1-albumin adducts in plasma or serum, which are formed with the lysine amino acid of albumin, is used as a biomarker of AFB1. In the papers reviewed, AFB1-lys was the most commonly analyzed biomarker (29.9%); this was followed by OTA (21.6%), in both single- and multi-mycotoxin analyses. Table 3 provides a record of the biomarkers of mycotoxins in plasma and serum in the papers reviewed. These data are summarized in Figure 5.

#### 2.3.1. Single-Biomarker Studies.

Due to the complexity of the matrices and the physicochemical diversity of mycotoxins, most of the methods currently used to analyze these toxins in human plasma and serum samples focus on assaying one mycotoxin or some structurally related mycotoxins belonging to a single family, such as AFs (AFB1, AFB2, AFG1, AFG2, AFB1-lys) [49,57,64,65,75,76,77,78,79,81,82,86,87,88,89,90,91,92,112,113,114,115,116,117], OTA and OTα [54,55,61,62,63,85,118,119], CIT and DH-CIT [23,54,55,56] and ZEA and its metabolites [58,68,69].

Regarding AFs, the biomonitoring of plasma and serum is carried out analyzing AFB1 or by determining AFB1-albumin adducts. Albumin adducts are chosen primarily because they present a half-life of around 2–3 months and, hence, the presence of these adducts in plasma and serum samples indicates long-term and chronic exposure to AFB1. In addition, AFB1-albumin adducts are stable in serum samples stored at −80 °C for over 25 years, and can therefore be re-analyzed years later [120].

AFs in human body fluids have been studied less extensively than AFs in food, due to the lack of specific antibodies [37]. However, a specific monoclonal antibody for AFB1-lys has been developed (IIA4B3); thus, AFB1-lys measurement has proven to be more accurate than that of AFB1- albumin [121]. In addition, the use of labeled AFB1-^13^C_6_-^15^N_2_-lysine [49] or AFB1-D_4_-lys [113] as internal standards sometimes enhances the reliability of the method [122]. To release AFB1-lysine (AFB1-lys) adduct from albumin, proteins in serum samples are digested with Pronase^®^ for a few hours at 37 °C. AFB1-lys is then extracted and purified with SPE by means of a mixed-mode anion exchange reversed-phase matrix. Next, the analytes are eluted, mainly through the use of methanol (MeOH) with formic acid, then concentrated and reconstituted in the mobile phase before analysis with LC-MS/MS [49,65,81,83,87,112,113,114,115], LC-FLD [81,88,89,90,91,92] or ELISA [73,74,75,76,77,78,79,117]. McCoy et al. (2008) [70] and Scholl et al. (2006) [123] reported good correlations of the concentration levels obtained from ELISA, LC-FLD and LC-MS. These authors suggested that the overestimation they sometimes encountered when using the ELISA method was due to the detection of other aflatoxin metabolites that were co-extracted along with AFB1-lys adducts.

Moderate-to-high levels of AFs in plasma and serum were reported globally, depending on the sampling country and the LOD of the methods used (Table 3). Cross-sectional surveys were conducted among the US [124] and Kenyan population [125]. The US study reported much lower AF levels than those of developing countries, where aflatoxin exposure has become a public health problem.

In recent years, several studies have been carried out to identify a link between plasma or serum AF levels and certain diseases, including hepatocellular carcinoma (HCC), chronic cirrhosis, chronic hepatitis B, colorectal cancer, liver cancer and human immunodeficiency virus [57,73,74,112,126]. Koshiol et al. (2017) [87] found a relationship between aflatoxin exposure and gallbladder cancer (GBC) based on levels of AFB1-lys in plasma samples from Shanghai. The AFB1-lys adduct was detected in 32% of the patients with this pathology (5.4 pg/mg albumin) and 15% of the control group (1.2 pg/mg albumin). Díaz-León et al. (2019) [81] reported exposure to AFB1-lys in a high number of the samples analyzed from healthy women, and observed a high correlation between AFB1 exposure and some markers of renal injury. In addition, Jolly et al. (2015) [126] conducted a study in Kumasi (Ghana) in which they detected high levels of AFB1-lys in the blood of study participants that varied according to season. The authors concluded that stored food was most probably the source of AFB1 exposure. In Taiwan, two case-control studies were performed in order to investigate the risk associated with AFB1 in HCC patients with different forms of hepatitis (B or C). These studies reported that high exposure to AFB1 increases the risk of HCC in populations with risk factors for cirrhosis, such as alcohol consumers and/or hepatitis patients [73,74].

The effect of aflatoxin exposure in vulnerable groups, such as pregnant women and children, is of particular concern [127]. Hernandez-Vargas et al. (2015) [75] established an association between AFB1 exposure in pregnant women and DNA methylation, which may have an impact on the health of their children. Based on the possible association between infant exposure and poor growth effects and on previous findings that have demonstrated that AFs cross the placental barrier [128,129], Lauer et al. (2018) [90] studied the association between maternal aflatoxin exposure during pregnancy and adverse birth outcomes. AFB1-lys was detected in 100% of serum samples from the expectant mothers, with values ranging from 0.71 to 95.60 pg/mg albumin. Elevated levels of AFB1-lys were associated with lower infant birth weight, lower weight for age and smaller head circumference for age in infants at birth. Moreover, the metabolism of AFs to epoxides may increase during pregnancy. Groopman et al. (2014) [129] concluded that the fetus has the capacity to metabolize aflatoxin to levels comparable to those of the mother and, consequently, pregnancy may pose a high risk of aflatoxin exposure for pregnant women and their fetuses.

A number of studies have been carried out to evaluate the presence of AFs in plasma or serum from children with severe acute malnutrition (SAM) in Kenya [92], Nigeria [49], Tanzania [113,117] Uganda [88,90], Mexico [81,91] and other countries [75,77,78,79,83,87,114,115]. This is a particularly relevant topic due to the rates of childhood growth disorders in these countries. McMillan et al. (2018) [49] improved the accuracy of AFB1-lys quantification by means of a sample treatment that involved protein precipitation prior to Pronase^®^ digestion, followed by SPE cleanup; they also used ^13^C internal standards and LC-MS/MS. The authors used this methodology to evaluate the presence of AFB1-lys in Nigerian children suffering from SAM. AFB1-lys levels in all children (control and SAM) ranged from 0.2–59.2 pg/mg albumin and were significantly higher in children with SAM (4.3 pg/mg albumin) compared to controls (0.8 pg/mg albumin) (p-value: 0.0083). Leroy et al. (2018) [91] studied the association between serum AFB1-lys levels and height or height-for-age difference at several ages of children that were exposed to a milk-based multiple micronutrient-fortified food in Mexico. Low aflatoxin exposure was associated with greater child linear growth. In contrast, Chen et al. (2018) [113] studied plasma samples from children at 24 months of age (n = 60) in Tanzania and analyzed them for AFB1-lys. Seventy-two percent of the children had detectable levels of AFB1-lys, with a mean level of 5.1 pg/mg albumin; however, no association was observed between aflatoxin exposure and growth impairment. Another study linked aflatoxin exposure to chronic hepatomegaly, which could represent a health risk for children [130].

HBM is also useful for assessing possible occupational exposure to AFs [86]. Saad-Hussein et al. (2016) [76] observed higher levels of AFB1-albumin in plasma from bakers compared to milling workers, and in both groups of workers compared to the control group. The authors attributed this to the exposure to different AFs in the workplace. Ferri et al. (2017) [86] studied levels of AFB1, AFB2, AFG1, AFG2 and aflatoxin M1 (AFM1), the most prevalent metabolite of AFB1, in mill workers exposed to contaminated dust. These authors combined a cleanup step that used a specific IAC and detection with an LC-FLD method to achieve good recoveries (84%–111%) for all AFs, except for aflatoxicol (AFOH) (60%). While there was no quantifiable presence of free AFs, AFB1-adducts were detected after treatment with Pronase^®^.

All of these studies reinforced the idea that aflatoxin exposure is an important public health concern [81], and that HBM is a useful tool for studying the implication of AFs in different diseases [131]. In addition, they corroborate the premise that AF-adducts are the main biomarkers of AFs in plasma or serum.

With respect to OTA, direct detection in human plasma was widely performed. Because OTA binds to plasma proteins rapidly and with a high affinity, it constitutes a good biomarker of exposure due to its persistence in blood [17,24]. A good strategy for investigating human OTA exposure involves analyzing OTA levels in plasma or serum and converting internal OTA levels to estimated daily intake (EDI) values by means of the Klaassen equation (Equation (1)) [85,132]. The methodologies for OTA single-detection may include LLE or SPE of plasma or serum samples and analysis by LC-FLD [54,55,85,118] or by LC/MS/MS [61,62,63]. Some authors studied human whole blood samples instead of plasma or serum matrices. These samples were spotted, dried and extracted with a solvent consisting of acetone, ACN and water. The authors then analyzed the extract by LC-MS/MS. Using this methodology, Sueck et al. (2019) [63] and Cramer et al. (2015) [62] studied the presence of OTA and 2´R-OTA (an OTA isomer that appears during the roasting process) in the blood of coffee drinkers. The authors reported the long-term persistence of the OTA isomer in blood and higher levels in coffee drinkers; consequently, 2´R-OTA could represent a good biomarker of OTA ingestion in these cases.

The relation between OTA plasma levels and specific disease risk markers, such as body mass index [133,134], kidney disease and inflammation (C-reactive protein)[119], was investigated. Di Giuseppe et al. (2012) [119] observed a positive association between OTA intake, C-reactive protein and cardiovascular risk. Despite the fact that OTA was detected in nearly all plasma samples analyzed in the studies reviewed, the value of 500 ng/L, which was related to the onset of kidney diseases, was exceeded in very few. Prati et al. (2016) [118] carried out a study to correlate OTA serum levels and liver function in two groups (one with and the other without chronic liver disease). The authors concluded that there was a relationship between OTA and C-reactive protein that confirms its inflammatory effect. However, the authors recommended further studies on this topic be carried out to clarify whether OTA is a risk factor for HCC or cirrhosis. In particular, OTA could pose a risk to people suffering from liver disease due to its capacity to induce DNA damage. In addition, OTA has a fairly long half-life in human blood and accumulates in the kidneys [135].

Ali et al. (2018) [55] and Malir et al. (2019) [54] carried out studies on plasma samples to assess OTA exposure in humans. OTA and OTα were determined through validated LC-FLD approaches that involved cleanup with LLE, with or without the cleavage of conjugates. OTA was detected in 100% and 48% of the samples, at levels of 0.72 and 0.14 ng/mL, respectively. The biomarker-based intake estimates (1.44 and 0.29 ng/kg.bw for both studies, respectively) fell well below the health-based guidance value (HBGV) (14 ng/kg.bw) [136]. In the study of Malir et al. (2019) [54], the authors also explored if OTA may contribute to kidney diseases such as renal tumors. However, there were non-significant differences between the OTA levels in Czech plasma samples from healthy volunteers and those from renal tumor patients.

Woo et al. (2016) [85] analyzed OTA in serum samples from pregnant women and extrapolated the values obtained to the fetus. The authors considered EDI values for the fetus to be double those of the expectant mother. Although fetal exposure to OTA can be considered a high risk, limited information is available on such exposure and the associated toxicity problems.

In the case of ZEA, data suggesting a significant effect of exposure of ZEA metabolites on human health outcomes are scarce. Some studies have evaluated the relation between the presence of ZEA and its metabolites in biological samples with changes in estrogenic activity and adverse effects in the reproductive system. In a previous study, no differences between ZEA levels in plasma from control and cancer patients (cervical and breast cancer) was found [137]. Recently, Mauro et al. (2018) [68] studied the presence of ZEA and its metabolites in serum samples by performing enzymatic treatment of samples with glucuronidase, followed by purification through SPE columns and analysis by LC-MS/MS. The authors applied this methodology to samples from overweight or obese women in the USA. ZEA was detected in all obese women and exposure was associated with food intake (especially meat) and body mass index. The alteration of ZEA levels in the case of obese women, and consequently its potential implication for health, should be considered. Because there is evidence on endocrine disruption, particularly during fetal development, Fleck et al. (2016) [69] in USA, using a similar methodology, only detected ZEA in 1/11 serum samples from pregnant women, selected because of the presence of ZEA in the urine of the same individuals. Moreover, this positive serum sample had a level of ZEA near the limit of quantification (LOQ). In addition, a possible association with autism spectrum disorder (ASD) and ZEA exposure has been studied in Italy by De Santis et al. (2019) [58]. No ZEA values over the LOQ were obtained in serum samples from autistic children or from healthy controls.

CIT and its metabolite dihydrocitrinon (DH-CIT) in plasma have been described as good biomarkers of this mycotoxin in HBM [56]. Due to its nephrotoxicity, CIT may be a contributing factor to the high frequency of renal tumors in some countries [54]. The methods developed by Ali et al. (2019, 2018) [55,56], Malir et al. (2019) [54] and Degen et al. (2018) [23] to measure CIT and DH-CIT by means of an ACN protein precipitation, followed by centrifugation and analysis by LC-MS/MS, have been applied in studies on human plasma [23,55]. CIT was detected in almost all samples analyzed. However, due to the short half-life of this mycotoxin in human blood (about 9 h) and urine (6.7 h), and because CIT is extensively converted into DH-CIT (with a half-life of 8.9 h in urine), it does not accumulate in the organism and is considered to be of low concern in terms of human exposure, with the exception of some regions, including certain African countries, where further studies are required [56]. More biomarker-based analyses are needed to assess human health risks related to CIT exposure.

Biomonitoring data on other mycotoxins are very scarce and further research is required to gain more insight into their influence on human diseases.

Furthermore, humans suffer from exposure to multiple mycotoxins due to the fact that the human diet is varied and a number of fungi may be present in one raw material; therefore, the inevitable co-occurrence of different mycotoxins and their metabolites in human plasma and serum requires the development of a new approach to mycotoxin HBM.

#### 2.3.2. Multi-Biomarker Studies

Co-contamination is particularly significant due to potential additive, antagonistic and/or synergistic toxic interactions [18,138]. Controlling this simultaneous presence in the human body requires the development of analytical methods to analyze multiple mycotoxins in biological fluids [139]. In fact, this is the current trend in analytical method development. As described above, chromatographic systems coupled with tandem MS have become the reference technique in this research and were used in 90% of all articles reviewed that included multi-mycotoxin analysis. As with single-mycotoxin monitoring, the main ionization sources employed were ESI and APCI. Regarding mass analyzers in multi-biomarker studies, QqQ and QTrap were the most commonly used, at 50% and 30%, respectively.

To date, only a few methods have been developed for multi-analyte detection of mycotoxins in plasma and serum samples [48,53,58,59,60,64,65,82,111], and 90% of these methods have been developed in the last three years. Only five (50%) of these studies proposed methods for detecting more than 10 mycotoxins simultaneously. Additionally, some authors indicated that the LOQs obtained in these studies were not low enough for HBM studies [48], although no guidelines are available on this topic, as explained above (Section 2.2.).

The most common extraction procedure among the multi-detection methods reviewed was LLE (or SPE after an enzymatic procedure). This is due to the need for non-selective extraction procedures. Because sample pretreatment is critical, some authors assayed different preparatory methodologies to overcome the limitations presented by commonly used methodologies. For example, Osteresch et al. (2017) [59] proved that greater centrifugal force produces higher signal intensity and good limits of quantification in the lower pg/mL range for all 27 mycotoxins analyzed in serum samples. However, they reported high matrix effects. Slobodchikova and Vuckovic (2018) [48] used LC-HRMS (Orbitrap) to study different sample preparation procedures in order to reduce matrix effects, obtain a method for the multi-detection of 17 mycotoxins in human plasma and monitor the presence of unknown mycotoxins and metabolites. They selected three-step LLE, with which they obtained good recovery data. The low-cost method minimizes matrix effects, but is not suitable for OTA, FB1 or FB2 quantification. Arce-López et al. (2020) [66] recently developed and validated a LC-MS/MS methodology for the simultaneous analysis of 19 mycotoxins in plasma. Sample deproteinization and cleanup were performed in a single step by means of Captiva^®^ EMR-Lipid cartridges and several samples were processed simultaneously. Good recovery and matrix effect values were obtained due to the elimination of phospholipids from plasma during sample preparation and the use of matrix-matched calibration curves. LOD values ranged from 0.04 to 2.7 ng/mL (except for NIV) and mean recovery values from 68.8% to 97.6% (RDS ≤ 15%). The matrix effects were not significant for most of the mycotoxins and ranged from 75.4% and 109.3% (RDS ≤ 15%).

Some of the methods developed for multi-mycotoxin determination were designed to find a correlation between mycotoxin levels in plasma or serum and diseases, countries, seasons, gender, age and health statuses of donors (Table 3).

Fan et al. (2019) [65] studied multi-mycotoxin exposure in a rural population in China by means of a validated LC-M/MS method. Twelve mycotoxins were measured in plasma from 260 adults; 149 males and 111 females. In this study, OTA was the most abundant mycotoxin (27.7%). The EDI of OTA (2.4 ng/kg.bw/day) was lower than the tolerable daily intake (TDI) value, and the authors suggested that the potential health risk associated with this mycotoxin was low. Although the incidence and concentration of mycotoxins in males and females differed slightly, differences in mean concentrations between the two groups were not significant for all mycotoxins. More than 60% of the participants were exposed to one or more mycotoxin [65].

Ouhibi et al. (2020) [53] developed a method for detecting PAT, for the first time, and CIT in biological fluids to assess human exposure. Positive mycotoxin values were detected in 26% (PAT) and 36% (CIT) of the plasma samples from Tunisian participants, but no significant differences were observed between the control and colorectal cancer patients. In addition, higher CIT levels were detected in this study compared to previously published studies.

Cao et al. (2018) [64] developed a multi-mycotoxin method for analyzing 11 carcinogenic mycotoxins in plasma from patients with HCC, including enzymatic de-conjugation with β-glucuronidase. Moreover, despite the fact that the authors obtained high recovery values with MeOH/acetic acid, they ended up opting for deproteinization with ACN/acetic acid, since lower matrix effects were observed. Sixty samples (30 control and 30 HCC patients) were collected for the study. AFB1 and STER were the most prevalent mycotoxins and were detected more frequently in patients with chronic liver disease (33% and 40%, respectively) than in control patients (13%). The authors suggested that it would be useful to study the possible influence of STER on HCC, since levels were higher in ill people than in controls. AFB2 was detected in 12% of the samples. AFG1, AFG2, AFM1, CIT and OTA were detectable only at the LOD. No PAT levels were found in the samples.

In a cross-sectional study, De Santis et al. (2017) [82] determined mycotoxins in serum samples from children with autism and healthy controls. After the digestion of samples with Pronase^®^, purification with QuEChERS and an UHPLC-MS/MS analysis, the recoveries obtained were below 63%, except for AFB1 (82%). LOD ranged from 0.01 ng/mL for AFB1 to 11 ng/mL for gliotoxin (GLIO). In 2019, De Santis et al. [58] determined GLIO and OTA levels by means of UHPLC equipped with an FLD detector. Optimized recovery values for GLIO and OTA were obtained (63% and 75%). These studies sought to test the association between mycotoxins and ASD, since many gastrointestinal, inflammatory and neurological symptoms induced by mycotoxin exposure are similar to those often associated with ASD. Interestingly, these authors reported that children with autism have significantly lower levels of OTA in plasma when compared to their siblings and other healthy children. This could be explained by the altered OTA biotransformation pathway. Moreover, GLIO values in children were obtained for the first time.

A study was conducted in 2018 to analyze exposure to AFB1 and other mycotoxins among workers at a waste-sorting plant [60]. AFB1, enniatin B (EnB) and OTA, as well as 2’R-OTA, were detected and quantified through a multi-mycotoxin LC-MS/MS approach. The authors concluded that this study confirmed co-exposure and different possible exposure routes.

Finally, Ens (EnA, EnA1, EnB and EnB1) and BEA were determined through LC-MS/MS by Serrano et al. (2015) [111]. They achieved good recoveries (90%–120%) and LODs ranging from 10 ng/L for EnA1 to 40 ng/L for BEA.

### 2.4. Risk Characterization

Toxicokinetic data are very useful for human health risk assessment. Unfortunately, in the field of mycotoxins, these data are very scarce in humans. Related to half-life values, they have only been established for some of the toxins. CIT has a short half-life in human blood of 9.4 h [56], whereas, for OTA, a long half-life of 35.6 days has been described [135] due to its high binding to plasma proteins [140]. For ZEA, these data are still unknown [141], although Mukherjee et al. (2014) [142], using a physiologically-based toxicokinetic model, estimated a half-life for ZEA of 11.89 h in young girls. The half-life for AFB1 in four human volunteers was 64.4 h [143]; although it should be taken into account that authors did not discriminate between AFB1 and its metabolites or conjugates [144]. For AFB1-lys, and due to lysine stability in human serum, a half-life of 2–3 months was estimated by Mupunga et al. (2016) [145]. These authors also stated that AFM1 has a short half-life, though no value was indicated. No information about the human toxicokinetics of DON and its derivatives, T-2, HT-2, FUS-X, NIV [45], Ens and BEA [146] or diacetoxyscirpenol (DAS) [147] was found.

The health risks associated with mycotoxin exposure arise from their toxicity. To minimize the risk to human health, several international bodies, such as the European Food Safety Authority (EFSA) or the Joint FAO-WHO Expert Committee on Food Additives (JECFA), have carried out health risk assessments for mycotoxins (or group of mycotoxins) (Table 4) and have established health-based guidance values (HBGV), such as the TDI. Due to the carcinogenic risk associated with some of the mycotoxins, the International Agency for Research on Cancer (IARC) has also evaluated and has classified some of them as: i) carcinogenic to humans (Group 1), based on sufficient human data; or ii) possibly carcinogenic to humans (Group 2B), based on sufficient experimental/animal data but limited human epidemiological information. Due to the lack of experimental data or epidemiological information at the time in which the IARC evaluation was carried out, many of the mycotoxins were also classified as Group 3 (not classifiable as to its carcinogenicity to humans). It should be mentioned that for compounds known to be genotoxic and carcinogenic, such as AFs or STER, the general assessment is that exposure from all sources should be as low as is reasonably achievable. Indeed, in these cases, EFSA did not consider it appropriate to establish HBGV and therefore proposed the margin of exposure (MOE) approach in their risk assessments.

Health risk can be evaluated by means of EDI through food consumption data/exposure data and/or biomarker levels in human plasma/serum. The exposure related-data obtained are then compared to the TDI established for the mycotoxin (or group) under study [22,148]. A strategy that combines both approaches can also be adopted [40].

In the papers reviewed, OTA and AFB1-lys were the most frequently detected biomarkers in plasma and serum samples. They reported positive levels of 64.9% and 76.9% of the samples analyzed for these mycotoxins, respectively. The samples analyzed were taken from documented patients with different diseases (SAM, HCC, GBC and ASD) and from control individuals. Therefore, although low levels were detected, it is possible to conclude that the world population in general is exposed to AFB1 and OTA. Assessing the risk posed by both mycotoxins is thus of great interest.

The correlation between OTA concentration in plasma, *C_p_* (ng/mL), and the estimated daily intake (EDI), expressed as *ko,* can be calculated by means of the Klaassen equation (equation 1) [85,132] where *Cl*_renal_ is the daily renal clearance and *A* refers to OTA bioavailability:

(1) ko (ng/kg.bw/day)=Clrenal CP A

After considering several assumptions to express the equation based on human data (plasma clearance of 0.99 mL/kg of body weight/day and an estimated *A* value of 0.5), the Klaassen equation can be expressed as follows [33,118]:

(2) $$EDI=1.98\;\times \;C_{P}$$

**Figure 6:**
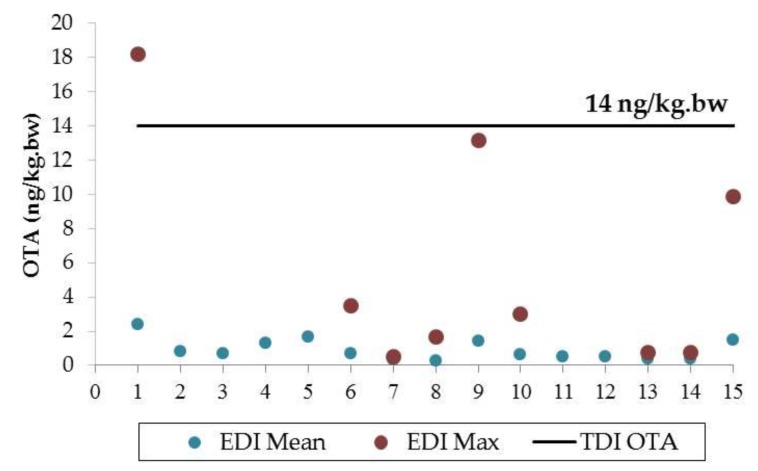
Comparison between estimated daily intake (EDI) of mean and maximum values and TDI for OTA exposure. Each dot corresponds to the EDI mean (blue) or EDI max (red) for the different exposure studies retrieved for OTA. A total of 15 studies were evaluated. The black line represents the most recent TDI established for OTA [136].

According to the studies included in this review, it should be noted that mean OTA concentrations in blood samples did not vary considerably (around 1 µg/L) across the world (Table 3). EDIs were calculated based on Equation (2) using the mean and maximum OTA levels observed in studies that addressed OTA detection. Based on the mean OTA concentration, the calculated EDI values ranged from 0.28 to 2.40 ng/kg.bw/day (Figure 6), which were considerably lower than the TDI for OTA (14 ng/kg.bw/day). Only one of the maximum values detected (9.18 µg/L) [71] led to an EDI value higher than the TDI established. Therefore, although positive OTA levels were observed in 64.9% of the total plasma and serum samples analyzed, the EDI values obtained did not exceed the risk value established for human health.

AFB1 is a known carcinogenic agent in humans (Group 1) and can lead to growth suppression, immune system modulation and malnutrition, even at low concentrations [27,161]. Intake of a small amount, such as 1 ng/kg.bw day, is considered dangerous and toxic for human health [22]. This is of particular concern in children, since lower values than those indicated for adults can have lethal consequences [162]. Therefore, the measurement of AFB1 and/or its metabolites in biological matrices is crucial for assessing potentially dangerous exposure to this toxin.

The correlation between AFB1-lys levels in plasma or serum and dietary exposure to AFB1 was investigated in the literature. It was calculated that around 1.4–2.3% of ingested AFB1 is covalently bound to albumin [37]. Moreover, a strong correlation coefficient of 0.80 between AFB1-lys levels in serum or plasma albumin and dietary exposure to AFB1 has been reported [163]. For AFs, however, no clear relationship between plasma concentration and EDI has been established [65], a fact that has made it impossible to calculate the EDI value [144].

The AFB1 adduct concentrations (pg/mg albumin) reported by the papers reviewed were as follows: mean values ranged from 0.8 to 31.2, and maximum values ranged from 0.10 to 211. The worst scenario was observed in studies carried out in developing countries. The lowest values were those found in developed countries and in some control samples in Nigeria [49] and China [87].

To improve risk assessment, further studies should be carried out to monitor rarely studied toxins such as T-2 and HT-2, as well as other forms in which mycotoxins are found, such as glucuronide conjugates [22].

## 3. Conclusions

Mycotoxins are recognized as toxic compounds of great concern in the context of human health and the global economy. HBM of mycotoxin biomarkers is considered a good approach to obtain data that could help determine human exposure, assess risks and identify relationships between diseases and mycotoxins. This creates new challenges in the field of mycotoxin research. In the present review, some of these challenges, together with other related aspects, have been identified.

Good biomarkers for each mycotoxin of interest should be described, including the ones for the so-called modified mycotoxins. Consequently, studies on the metabolism and toxicokinetics of mycotoxins are of vital importance. In addition, validated analytical methods should be developed. The current analytical trend is to simultaneously detect multiple mycotoxins in a single run with a view to saving time and reducing costs. The LC methodology coupled to several detectors, especially MS/MS and HRMS, has proven to be a useful analytical technique for multi-mycotoxin biomonitoring. The development of these methods requires adequate and, if possible, affordable standards and reference materials. New methodologies for sample treatment that reduce matrix effects are needed. Finally, guidelines for the validation of analytical methods should also be developed.

AFB1-lysine and OTA in plasma and serum have been the most widely studied biomarkers in recent years. For AFB1-lys detection, most articles proposed digestion with Pronase^®^ and a purification step by means of SPE before analysis with LC coupled to FLD, MS/MS or HRMS detectors. AFs such as AFB1, AFB2, AFG1, AFG2 and AFM1, CIT and ZEA have also been analyzed, but to a lesser extent. Mycotoxins such as T-2 and HT-2 were not studied in the papers reviewed.

A high percentage of the samples analyzed for OTA and AFB1 (mostly as AFB1-lys) presented some level of these mycotoxins; it can therefore be concluded that the general population is exposed to them. In the case of OTA, the EDI values calculated, based on either the mean or maximum values, were, in all cases, lower than the TDI value defined for this mycotoxin. For the other mycotoxins, equations that relate biomarkers of exposure and concentration in biological fluids or tissues need to be determined. This aspect is crucial for improving knowledge and the interpretation of the results obtained through HBM.

Several authors have searched for a relationship between diseases and mycotoxin levels in plasma or serum. Some relationships have been detected, but others remain unclear. It is not even clear whether the presence of mycotoxins in biological samples is the cause (or a contributor, along with other factors) of a disease, or, on the contrary, whether its presence is the result of the metabolism pathway alteration produced by the illness itself, which could increase mycotoxin levels in ill people.

Based on all of the above, it is possible to conclude that new avenues are emerging, and much more research is required on the interesting and important topic of mycotoxin HBM.

## 4. Materials and Methods

**Figure 7:**
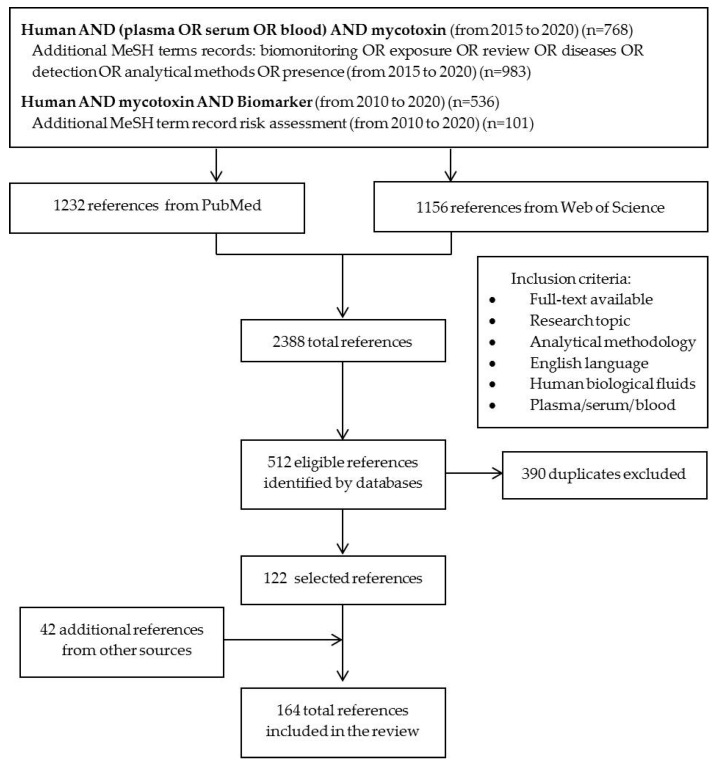
Flow diagram of excluded and included studies based on PRISMA Statement. MeSH: Medical Subject Headings.

A systematic review strategy was carried out based on PRISMA Statement [164]. For this purpose, the PubMed and Web of Science databases were used. First, two general searches were performed with the aim of obtaining a general overview of the subject, as shown in Figure 7 (bold). Additional searches were then carried out with more specific medical subject heading (MeSH) terms: “biomonitoring” OR “exposure” OR “review” OR “disease” OR “detection” OR “analytical methods” OR “presence” (added to the first general search); and “risk assessment” (added to the second search). A total of 2388 articles were obtained with this search. Inclusion criteria were: full-text was available, papers addressed the research topic, papers described analytical methodologies, articles were written in English and articles focused on human biological fluids, especially plasma, serum and blood. Articles that did not meet these criteria were excluded, as well as duplicated records. Other sources were also used, such as the reference section of the papers reviewed, the IARC, the European Commission and the EFSA. In total, 164 articles were evaluated (Figure 7).

## Figures and Tables

**Table 1:** Chemical characteristics of the studied analytes in the retrieved articles.

Biomarker	Molecular Formula	CAS Number	Molar Mass (g/mol)	Log P	Water Solubility (25 °C) (mg/L)
	C_17_H_12_O_6_	1162-65-8	312.4	1.23^b^	16.14^b^
AFB1					
AFB2	C_17_H_14_O_6_	7220-81-7	314.3	1.45	24.9
AFG1	C_17_H_12_O_7_	1165-39-5	328.3	0.5	477
AFG2	C_17_H_14_O_7_	7241-98-7	330.3	0.71	3.73 × 10^3^
AFM1	C_17_H_12_O_7_	6795-23-9	328.3	1.21^a^	0.99 g/L^a^
AFM2	C_17_H_14_O_7_	6885-57-0	330.3	1.16^a^	2.16 g/L^a^
AFOH	C_17_H_14_O_6_	29611-03-8	314.3	1.19^b^	0.56 g/L^b^
ALT	C_15_H_16_O_6_	29752-43-0	292.3	1.12 ^c^	5692 ^c^
AME	C_15_H_12_O_5_	23452-05-3	272.2	2.25^b^	0.09 g/L^b^
AOH	C_14_H_10_O_5_	641-38-3	258.2	2.49^a^	0.23 g/L^a^
BEA	C_45_H_57_N_3_O_9_	26048-05-5	783.9	5..25^b^	0.00088 g/L^b^
CIT	C_13_H_14_O_5_	518-75-2	250.2	0.45	Practically insoluble
DH-CIT	C_13_H_14_O_6_	65718-85-6	266.2	3.61 ^c^	59.67 ^c^
DAS	C_19_H_26_O_7_	2270-40-8	366.4	1.40^a^	1.03 g/L^a^
DOM-1	C_15_H_20_O_5_	88054-24-4	280.3	0.16 ^c^	2.17 × 10^4c^
DON	C_15_H_20_O_6_	51481-10-8	296.3	0.71	5.5 × 10^4^
3-ADON	C_17_H_22_O_7_	50722-38-8	338.4	0.61^a^	5.99 g/L^a^
15-ADON	C_17_H_22_O_7_	88337-96-6	338.4	0.54^a^	6.31 g/L^a^
DON-3-GlcA	C_21_H_28_O_12_	1000000-13-4	472.4	n.i.	n.i.
DON-15-GlcA	C_21_H_28_O_12_	1372859-16-9	472.4	n.i.	n.i.
EnA	C_36_H_63_N_3_O_9_	2503-13-1	681.9	4.79^b^	0.011 g/L^b^
EnA1	C_35_H_61_N_3_O_9_	4530-21-6	667.9	4.39^b^	0.012 g/L^b^
EnB	C_33_H_57_N_3_O_9_	917-13-5	639.8	3.81^b^	0.018 g/L^b^
EnB1	C_34_H_59_N_3_O_9_	19914-20-6	653.8	4.06^b^	0.018 g/L^b^
FB1	C_34_H_59_NO_15_	116355-83-0	721.8	0.81^a^	0.043 g/L^a^
FB2	C_34_H_59_NO_14_	116355-84-1	705.8	0.28^a^	0.015 g/L^a^
FUS-X	C_17_H_22_O_8_	23255-69-8	354.4	1.24	6.67x10^4^
GLIO	C_13_H_14_N_2_O_4_S_2_	67-99-2	326.4	0.36^b^	12.9 g/L^b^
HT-2	C_22_H_32_O_8_	26934-87-2	424.5	0.52^a^	1 g/L^a^
HT-2-4-GlcA	C_22_H_40_O_14_	1400867-48-2	600.6	n.i.	n.i.
NIV	C_15_H_20_O_7_	23282-20-4	312.3	2.24	3.54 × 10^5^
OTA	C_20_H_18_ClNO_6_	303-47-9	403.8	4.74	0.4246
OTB	C_20_H_19_NO_6_	4825-86-9	369.4	3.77	4.4
OTα	C_11_H_9_ClO_5_	19165-63-0	256.6	3.77 ^c^	49.35 ^c^
10-OH-OTA	C_20_H_18_ClNO_7_	86072-87-9	419.8	3.20 ^c^	n.i.
PAT	C_7_H_6_O_4_	149-29-1	154.1	0.27^a^	163 g/L^a^
STER	C_18_H_12_O_6_	10048-13-2	324.3	3.81	1.44
T-2	C_24_H_34_O_9_	21259-20-1	466.5	2.27	95.9
ZAN	C_18_H_24_O_5_	5975-78-0	320.4	4.86 ^c^	2.53 ^c^
ZAN-14-GlcA	C_24_H_32_O_11_	n.i.	496.5	n.i.	n.i.
ZEA	C_18_H_22_O_5_	17924-92-4	318.4	3.04^a^	0.12 g/L^a^
ZEA-14-GlcA	C_24_H_30_O_11_	1032558-19-2	494.5	n.i.	n.i.
α−ZΕL	C_18_H_24_O_5_	36455-71-7	320.4	3.27^a^	0.15 g/L^a^
β−ZΕL	C_18_H_24_O_5_	5916-52-9	320.4	3.27^a^	0.15 g/L^a^
α−ZAL	C_18_H_26_O_5_	26538-44-3	322.4	3.23^a^	0.16 g/L^a^
β-ZAL	C_18_H_26_O_5_	42422-68-4	322.4	3.23^a^	0.16 g/L^a^

n.i.: not indicated. AFB1: aflatoxin B1; AFB2: aflatoxin B2; AFG1: aflatoxin G1; AFG2: aflatoxin G2; AFM1: aflatoxin M1; AFM2: aflatoxin M2; AFOH: aflatoxicol; ALT: altenuene; AME: alternariol monomethyl ether; AOH: alternariol; BEA: beauvericin; CIT: citrinin; DH-CIT: dihydrocitrinone; DAS: diacetoxyscirpenol; DOM-1: deepoxy-deoxynivalenol; DON: deoxynivalenol; 3-ADON: 3-acetyldeoxynivalenol; 15-ADON: 15-acetyldeoxynivalenol; DON-3-GlcA: deoxynivalenol-3-glucuronide; DON-15-GlcA: deoxynivalenol-15-glucuronide; EnA: enniatin A; EnA1: enniatin A1; EnB: enniatin B; EnB1: enniatin B1; FB1: fumonisin B1; FB2: fumonisin B2; FUS-X: fusarenon-X; GLIO: gliotoxin; HT-2: HT-2 toxin; HT-2-4-GlcA: HT-2-4-glururonide; NIV: nivalenol; OTA: ochratoxin A; OTB: ochratoxin B; OTα: ochratoxin α; 10-OH-OTA: 10-hydroxyochratoxin A; PAT: patulin; STER: sterigmatocystin; T-2: T-2 toxin; ZAN: zearalanone; ZAN-14-GlcA: zearalanone-14-glucuronide; ZEA: zearalenone; ZEA-14-GlcA: zearalenone-14-glucuronide; α-ZEL: α-zearalenol; β-ZEL: β-zearalenol; α-ZAL: α-zearalanol; β-ZAL: β-zearalanol. Data extracted from: Hazardous Substances Data Bank (http://toxnet.nlm.nih.gov), except those indicated as ^a^Metabolomics Innovation Centre (https://www.metabolomicscentre.ca); ^b^Toxic Exposome Database (http://www.t3db.ca/) and ^c^ ChemSpider (http://www.chemspider.com/).

**Table 2:** Analytical methods for the analysis of mycotoxin biomarkers in human blood/plasma/serum samples.

Analyte/s	LOD (µg/L) ( *pg/mg Albumin)	Sample Preparation	Separation and Detection Technique	Year	Ref.
AFs (B1, B2, G1, G2, M1), OTA, OTB, T-2, HT-2, DOM-1, ZEA, STER NIV, DON, 3-ADON, 15-ADON, NEO, FUS-X, DAS	0.04–2.7 0.15–9.1	**SPE** 400 µL plasma + 1.2 mL ACN (1% formic acid) + SPE (Captiva® EMR-Lipid cartridges). Evaporate to dryness (60 °C) + 200 µL MeOH/H_2_O with 5 mM ammonium formate (40:60) **SPE** 400 µL plasma + 1.2 mL ACN (1% formic acid) + SPE (Captiva® EMR-Lipid cartridges). Evaporate to dryness (60 °C) + 200 µL MeOH/H_2_O with 5 mM ammonium formate (5:95)	**LC-MS/MS** Column: C18 (150 × 2.1 mm × 2.7μm) at 45 °C Flow: 0.4 mL/min Mobile phase: (A) MeOH (5mM ammonium formate with 0.1% formic acid); (B) H_2_O (5mM ammonium formate with 0.1% formic acid in 5:95 MeOH/ H_2_O) in gradient conditions Detector: ESI (+), QqQ, MRM	2020	[66]
PAT CIT	1.10 0.04	**QuEChERS** 1 mL plasma + 9 mL extraction solvent (53/44/3, ACN/ H_2_O /formic acid) + 2 mg MgSO_4_ + 0.5g NaCl. Shaking and extract using an agitator decanter (30 min). Centrifuge, evaporate supernatant to dryness (N_2_ and 40 °C) and reconstitute with 500 µL H_2_O /MeOH (90/10)	**LC-MS/MS** Column: C18 (100 × 2.1 mm × 1.8μm) at 45 °C Flow: 0.4 mL/min Mobile phase: (A) H_2_O /ACN (95.5/0.05); (B) MeOH/ACN (95.5/0.05) both with 5mM ammonium acetate in gradient conditions Detector: ESI (-), QqQ, MRM	2020	[53]
OTA, 2’R-OTA	0.006	**LLE** 100 µL dried blood spots + 10 µL d_5_-OTA i.s.+ 900 µL H_2_O/acetone/ACN (30:35:35). Sonicate. Evaporate (60 °C, reduced pressure). Reconstitute with 80 µL H_2_O/ACN/acetic acid (97:3:0.1)	**LC-MS/MS** Column: C18 (100 × 2.0 mm × 3 μm) at 40 °C Flow: 0.3 mL/min Mobile phase: (A) ACN (2% acetic acid); (B) H_2_O (0.1% acetic acid) in gradient conditions Detector: ESI (+), QTrap, MRM	2019	[63]
CIT	0.02	**LLE** 1 mL plasma + 1 mL ACN (1:1). Centrifuge. Evaporate (N_2_, 40 °C). Reconstitute in 350 µL MeOH. Filter	**LC-MS/MS** Column: C18 HD (125 × 3 mm) at 21 °C Flow: 0.2 mL/min Mobile phase (A) H_2_O; (B) MeOH both with 1mM ammonium formate in gradient conditions Detector: ESI (-), QTrap, MRM	2019	[54]
OTA	0.04	**IAC** 3 mL acidified serum + IAC column Ochraprep® (Biopharm)	**LC-FLD** Column: C18 (150 × 4.6 mm × 5 μm) Flow: 1.5 mL/min Mobile phase: MeOH/ ACN/ 0.05 mM sodium acetate/ acetic acid (300/300/400/14) Detector: 333/465 nm (λex/λem)	2019	[54]
AFs (B1, B2, G1, G2, M1, M2), OTA, OTα, FB1, T-2, HT-2 DON, 3-ADON, 15-ADON, DON-3-GlcA, DON-15-GlcA, FUS-X, ZEA, ZAN,α-ZEL, β-ZEL, α-ZAL, β-ZAL, ZEA-14-GlcA, ZAN-14-GlcA	0.03–0.5	**LLE** 200 µL plasma + 1 mL ACN/formic acid (99/1). Centrifuge, evaporate supernatant (N_2_) + 200 µL ACN/H_2_O with 5 mM ammonium acetate (20:80)	**LC-MS/MS** Column:C18 (100 × 3 mm × 2.7 μm) at 40 °C Flow: 0.4 mL/min Mobile phase: (A) MeOH; (B) H_2_O/ 5mM ammonium acetate in gradient conditions Detector: ESI (±), QTrap, MRM	2019	[65]
GLIO, OTA	0.05–25	**LLE** Serum + EtOAc. Evaporate (N_2_). Redissolve in MeOH/H_2_O (50:50), 2% acetic acid	**LC-FLD** Column: C18 (100 × 2.1 mm × 1.7 μm) at 40 °C Flow: 0.4 mL/min Mobile phase: H_2_O/MeOH (50:50), 2% acetic acid Detector: 333/460 nm (λex/λem)	2019	[58]
ZEA, α and β-ZEL	n.i	**LLE** Serum + 2 mL diethylether. Centrifuge. Evaporate (N_2_, 40 °C)	**LC-MS/MS** Column: (100 × 2.1 mm × 1.7 μm) at 40 °C Flow: 0.4 mL/min Mobile phase: H_2_O/MeOH (50:50), 2% acetic acid Detector: ESI (-), QqQ, MRM	2019	[58]
CIT, DH-CIT	0.02	**LLE** 750 µL plasma + 1 mL ACN. Centrifuge. Evaporate (N_2_, 40 °C). Reconstitute with 350 µL MeOH	**LC-MS/MS** Column: C18 HD (125 × 3 mm) at 30 °C Flow: 0.2 mL/min Mobile phase: (A) H_2_O; (B) MeOH both with 1mM ammonium formate in gradient conditions Detector: ESI (-), QTrap, MRM	2019 2018	[23,56]
NIV, DON, FUS-X, 3-ADON, 15-ADON, T-2, HT-2, AFs (B1, B2, G1, G2), ZEA, α-ZEL, β-ZEL, OTA, ZAN, α-ZAL, β-ZAL	0.04–1.5	**LLE** 100 µL plasma + 150 µL EtOAc. Centrifuge. Evaporate organic phase (dryness). Reconstitute in 200 µL MeOH	**LC-HRMS** Column: C18 (50 × 2.1 mm × 2.6 μm) at 30 °C Flow: 0.3 mL/min Mobile phase: (A) H_2_O; (B) MeOH/0.1% (or 0.02%) acetic acid in gradient conditions Detector: ESI (±), Orbitrap	2018	[48]
AFs (B1, B2, G1, G2, M1), STER, PAT, CIT, FB1, FB2, OTA	0.05–0.41	**LLE** 200 µL plasma + 50 µL β-glucuronidase (overnight 37 °C) + 1 mL ACN/acetic acid (99/1). Centrifuge. Evaporate (N_2_). Reconstitute in 200 µL ACN/H_2_O. Filter	**LC-MS/MS** Column: C18 (100 × 2.1 mm × 2.6 μm) at 40 °C Flow: 0.2 mL/min Mobile phase: (A) H_2_O (ammonium acetate, acetic or formic acid); (B) ACN (MeOH for FB1) in gradient conditions Detector: ESI (±), QqQ, MRM	2018	[64]
CIT, DH-CIT	0.07–0.15	**LLE** 1 mL plasma + 1 mL ACN. Centrifuge. Evaporate (N_2_, 40 °C). Reconstitute in 350 µL MeOH. Filter	**LC-MS/MS** Column: C18 HD (125 × 3 mm) at 21 °C Flow: 0.2 mL/min Mobile phase: (A) H_2_O; (B) MeOH both with 1mM ammonium formate in gradient conditions Detector: ESI (-), QqQ, MRM	2018	[55]
OTA, OTα	0.05	**LLE** 0.5 mL plasma + 100 µL hydrolysis buffer (pH 5) + 100 µL β-Gluc/ArylS enzyme (overnight, 37°) + 3 mL 1% NaHCO_3_ (+ H_3_PO_4_) + 2 mL chloroform/isopropanol (97:3). Centrifuge. Evaporate (N_2_, 45 °C). Reconstitute in 250 µL MeOH/H_2_O (1:1). Filter	**LC-FLD** Column: C18 (250 × 3 mm × 5 μm) at 40 °C Flow: 0.8 mL/min Mobile phase: (A) MeOH/2% acetic acid (66:34); (B) MeOH/isopropanol (90:10) in gradient conditions Detector: 333/450 nm (λex/λem)	2018	[55]
ZEA, α-ZEL, β-ZEL, ZAL, ZAN, β-ZAL	0.07	**SPE** 0.5 mL serum + 10 µL β-glucuronidase + 0.25 mL sodium acetate buffer (overnight, 37 °C) + 1 mL ChemElutTM cartridge. Elute with methyltertbutylether. Evaporate and redissolve in 35 µL of H_2_O/MeOH/ACN (2:1:1)	**LC-MS/MS** Column: C18 (50 × 2.1 mm × 1.9 μm) Flow: 0.2 mL/min Mobile phase: (A) MeOH; (B) H_2_O; (C) ACN in gradient conditions Detector: APCI (-), QqQ, MRM	2018	[68]
EnB, OTA, 2’R-OTA	0.01–0.04	**LLE** 100 µL dried serum spots + 1 mL H_2_O/acetone/ACN (30:35:35). Sonicate. Evaporate. Reconstitute with H_2_O/ACN/acetic acid (95:5:0.1)	**LC-MS/MS** Column: C18 (100 × 2 mm × 3 μm) at 45 °C Flow: 0.3 mL/min Mobile phase: (A) ACN (2% acetic acid); (B) H_2_O (0.1% acetic acid) in gradient conditions Detector: ESI (±), QTrap, MRM	2018	[60]
AFs (B1, B2, G1, G2, M1), ALT, AME, AOH, BEA, CIT, DH-CIT, DON, DON-3-GlcA, En (A, A1, B, B1), FB1, 10-OH-OTA, HT-2, HT-2-4-GlcA, OTA, 2’R-OTA, OTα, T-2, ZAN, ZEA	0.0012-1.34	**LLE** 100 µL dried blood or serum spots + 1 mL H_2_O/acetone/ACN (30:35:35). Sonicate. Evaporate (50 °C, low pressure). Reconstitute with H_2_O/ACN/acetic acid (95:5:0.1)	**LC-MS/MS** Column: C18 (100 × 2 mm × 3 μm) at 45 °C Flow: 0.75–0.85 mL/min Mobile phase: (A) ACN (2% acetic acid); (B) H_2_O (0.1% acetic acid) in gradient conditions Detector: ESI (±), QTrap, MRM	2017	[59]
AFB1, AFM1, DON, ZEA, DOM-1, FB1, GLIO, OTA	0.005-5.5	**QuEChERS** 1 mL serum + 1 mL PBS + Pronase®, (overnight, 37°) + 2 mL EtOAC (1% formic acid). Centrifuge, evaporate (N_2_) + 1 mL of ACN + 1.6 g QuEChERS (DisQUE®). Centrifuge, evaporate (N_2_) and reconstitute with 0.3 mL MeOH/H_2_O 3% formic acid and 5 mM ammonium formate (50/50)	**LC-MS/MS** Column: C18 (50 × 3 mm × 2.6 μm) at 40 °C Flow: 0.3 mL/min Mobile phase: (A) ACN/H_2_O (50/50); (B) MeOH/H_2_O (50/50) both with 5mM ammonium formate and 3% formic acid in gradient conditions Detector: ESI (+), QqQ, MRM	2017	[82]
AFB1, AFB2 AFG1, AFG2 AFM1, AFOH	0.006-0.025	**IAC** 5 mL serum + 400 µL PBS + IAC column (AFLAPREP®)	**LC-FLD** Column: C18 (150 × 2.1 mm × 5 μm) Flow: 1.2 mL/min Mobile phase: MeOH/H_2_O/ACN (20:20:60) (derivatizing agent: 100 µL HNO_3_ 65% and 119 mg KBr) Detector: 365/440 nm (λex/λem)	2017	[86]
ZEA	n.i.	**SPE** 100 µL serum + β-glucuronidase/sulfatase (24 h) + Novum SLE plate. Elute with methyltertbutylether. Evaporate and redissolve in 100 µL of H_2_O/MeOH (50/50)	**LC-MS/MS** Column: C18 (100 × 2.1 mm × 1.9 μm) at 50 °C Flow: 5 mL/min Mobile phase: (A) H_2_O; (B) ACN in gradient conditions Detector: ESI (±), QqQ, MRM	2016	[69]
OTA	0.2	**SPE** 0.5 mL serum + 15 µL acetic acid + SPE cartridge (Stata-C18). Elute with acidified MeOH (MeOH/acetic acid, 95/5). Evaporate and reconstitute with 0.5 mL MeOH	**LC-FLD** Column: C18 (250 × 4.6 mm × 5 μm) at 30 °C Flow: 1 mL/min Mobile phase: ACN/ H_2_O/ acetic acid (50/49/1) Detector: 310/465 nm (λex/λem)	2016	[85]
OTA, 2’R-OTA	0.006	**LLE** 100 µL dried blood spots + 1 mL H_2_O/acetone/ACN (30:35:35). Sonicate. Evaporate (60 °C, reduced pressure). Reconstitute with 100 µL H_2_O/MeOH/formic acid (60:40:0.1)	**LC-MS/MS** Column: C18 (150 × 2 mm × 5 μm) at 40 °C Flow: 0.3 mL/min Mobile phase: (A) MeOH; (B) H_2_O both with 0.1% formic acid in gradient conditions Detector: ESI (±), QTrap, MRM	2016 2015	[61,62]
AFB1, AFB2 AFG1, AFG2	0.025–0.05	**LLE** 1 mL serum + 2 mL hexane. Centrifuge. Supernatant + 1 mL chloroform. Shake. Centrifuge. Evaporate (N_2_) + derivatize with trifluoroacetic acid (TFA)	**LC-FLD** Column: C18 (250 × 3.8 mm × 5 μm) Flow: 1 mL/min Mobile phase: H_2_O/ACN/MeOH (62:16:22) Detector: 360/430 nm (λex/λem)	2015	[57]
Ens (A, A1, B, B1) and BEA	0.01–0.02	**SPE** 250 µL plasma + 25 mL MeOH/H_2_O (40:60) + Carbograph clean up	**LC-MS/MS** Column: C18 (150 × 2.1 mm × 3 μm) at 30 °C Flow: 0.75–0.85 mL/min Mobile phase: (A) H_2_O; (B) MeOH both with 5 mM ammonium formate and 0.1% formic acid in gradient conditions Detector: ESI (+), QqQ, MRM	2015	[111]
AFB1-lys	0.5	**Pronase® + SPE** 250 µL serum + Pronase® (5 h, 37°) + SPE column (Oasis® MAX). Elute with 2% formic acid in MeOH	**LC-MS/MS** Column: C18 (100 × 3 mm × 2.7μm) at 40 °C Flow: 0.3 mL/min Mobile phase: (A) MeOH; (B) H_2_O/0.1% formic acid in gradient conditions Detector: ESI (+), QqQ, MRM	2019	[65]
AFB1-lys	0.35	**Pronase® + SPE** 250 µL serum + Pronase® (4.5 h, 40°) +500 µL H_2_O + SPE column (Oasis® MAX). Elute with 2% formic acid in MeOH	**LC-FLD** Column: C18 (150 x 4.6 mm x 2.6 μm) at 25ºC Flow: 0.4 mL/min Mobile phase: (A) H_2_O/ MeOH (95:5) with 1% acetic acid; (B) MeOH/ H_2_O (95:5) with 1% acetic acid; (C) ACN in gradient conditions Detector: 370/470 nm (λex/λem) **LC-MS/MS** Column: C18 (50 × 2.1 μm × 2.7μm) at 40 °C Flow: 0.6 mL/min Mobile Phase: (A) H_2_O (0.06% formic acid); (B) ACN (0.06% formic acid) in gradient conditions Detector: ESI (+), TOF	2019	[81]
AFB1-lys	0.022	**Pronase® + SPE** 230 µL plasma + 805 µL MeOH:H_2_O (8:2). Centrifuge. Supernatant + 230 µL PBS + 13C i.s. + 230 µL Pronase® (overnight, 37°) + 460 µL H_2_O + SPE column (OASIS® MAX). Elute with 2% formic acid in MeOH	**nanoLC-HRMS** Column: C18 (75 µm × 15 cm) Flow: 0.3 mL/min Mobile phase: (A) H_2_O; (B) ACN both with 0.1% formic acid in gradient conditions Detection: nanospray, Orbitrap, full MS	2018	[49]
AFB1-lys	0.2–0.4^ *^	**Pronase® + SPE** 150 µL serum + Pronase® (3 h, 37°) + SPE column (OASIS® MAX). Elute with 2% formic acid in MeOH. Evaporate and dissolve in MeOH	**LC-FLD** Column: C18 (250 x 4.6 mm x 5μm) at 25 °CFlow: 1 mL/minMobile phase: (A) 20 mM NH_4_H_2_PO_4_;(B) MeOH in gradient conditionsDetector: 405/470 nm (λex/λem)	2019 2018 2016 2015	[89,90,91,92]
AFB1-lys	0.4–0.5^ *^	**Pronase® + SPE** 200 µL plasma + 10 µL x 0.1ng AFB1-D_4_-lys i.s. + Pronase® (18 h, 37°) + SPE column (OASIS® MAX). Elute with 2% formic acid in MeOH	**LC-MS/MS** Column: C18 (150 × 2 mm × 3μm) at 35 °C Flow: 0.25 mL/min Mobile phase: (A) H_2_O; (B) ACN; (C) 0.6% formic acid in gradient conditions Detector: ESI (+), QqQ, SRM	2019 2018 2017	[87,112,113,114,115]
AFB1-lys	6.0^ *^	**Pronase® + SPE** 250 µL serum + Pronase® (5 h, 37º) + SPE column (Oasis® MAX). Elute with 2% formic acid in MeOH. Evaporation. Dilute in 200 µL MeOH/H_2_O (25:75)	**LC-MS/MS** Column: C18 (50 × 2.1 mm × 1.7μm) at 40 °C Flow: 0.5 mL/min Mobile phase: (A) H_2_O; (B) ACN both with 0.1% formic acid in gradient conditions Detector: ESI (+), QqQ, MRM	2016	[83]
AFB1-alb	0.6–1.0^ *^	**Pronase® + SPE** 200 µg albumin + proteinase	**ELISA**	2018 2017 2015	[73,74,75]
AFB1-alb	2.5–3^ *^	**Pronase® + SPE** 2 mg albumin + proteinase (overnight) + Sep-Pak C18	**ELISA**	2018 2016	[77,78,79]
AFB1-alb	n.i	**IAC** EASI-Extract® Aflatoxin	**ELISA** Ridascreen® AFB1 30/15	2016	[76]

* pg/mg albumin. n.i: not indicated; 3-ADON: 3-acetyldeoxynivalenol; 15-ADON: 15-acetyldeoxynivalenol; 10-OH-OTA: 10-hydroxyochratoxin A; 2’R-OTA: 2’R-Ochratoxin A; ACN: acetonitrile; AFB1: aflatoxin B1; AFB1-alb: adduct of AFB1 with albumin; AFB1-lys: adduct of AFB1 with lysine; AFB2: aflatoxin B2; AFG1: aflatoxin G1; AFG2: aflatoxin G2; AFM1: aflatoxin M1; AFM2: aflatoxin M2; AFOH: aflatoxicol; AFs: aflatoxins; ALT: altenuene; AME: alternariol monomethyl ether; AOH: alternariol; APCI: atmospheric pressure chemical ionization; BEA: beauvericin; CIT: citrinin; DAS: diacetoxyscirpenol; DH-CIT: cihydrocitrinone; DOM-1: deepoxy-deoxynivalenol; DON: deoxynivalenol; DON-3-GlcA: deoxynivalenol-3-glucuronide; DON-15-GlcA: deoxynivalenol-15-glucuronide; EnA: enniatin A; EnA1: enniatin A1; EnB: enniatin B; EnB1: enniatin B1; Ens: enniatins; ESI: electrospray ionization; EtOAc: ethylacetate; FB1: fumonisin B1; FB2: fumonisin B2; FLD: fluorescence detector; FUS-X: fusarenon-X; GLIO: gliotoxin; HT-2: HT-2 toxin; HT-2-4-GlcA: HT-2-toxin-4-glucuronide; IAC: immunoaffinity columns; LLE: liquid-liquid extraction; LOD: limit of detection; MeOH: methanol; MRM: multiple reaction monitoring; NIV: nivalenol; OTα: ochratoxin α; OTA: ochratoxin A; PAT: patulin; PBS: phosphate buffer solution; QqQ: triple quadrupole; QTrap: quadrupole-ion trap; QuEChERS: Quick Easy Cheap Effective Rugged and Safe; SPE: solid-phase extraction; SRM: selective reaction monitoring; STER: sterigmatocystin; T-2: T-2 toxin; ZAL: zearalanol; ZAN: zearalanone; ZAN-14-GlcA: zearalanone-14- glucuronide; ZEA: zearalenone; ZEA-14-GlcA: zearalenone-14-glucuronide; ZEL: zearalenol.

**Table 3:** Studies on mycotoxin HBM.

Country	Analyte	Matrix	Total Samples	Positive Samples (%)	LOD LOQ (µg/L) or (pg/mg Albumin *)		Detection Technique	Mean (μg/L) and/or [Range] (μg/L or pg/mg Albumin *)	Year/Ref
Tunisia	PAT CIT	Plasma	50/50 50/50	20/30 34/38	1.10 0.04	2.30 0.09	LC-MS/MS	11.62^a^ 0.49 ^a^	2020 [53]
China	OTA FB1 DON ZEA ZAN	Plasma	260	27.7 2.7 2.3 6.5 1.2	0.04 0.2 0.5 0.05 0.03	0.1 0.51 0.1 0.1	LC-MS/MS	1.21 [0.312–9.18] 0.69 [0.305–0.993] 2.60 [1.39–5.53] 0.16 [0.063–0.418] 0.26 [0.164–0.346]	2019 [65]
Italy	GLIO OTA	Serum	110 (52/31/27) ASD/CS/C	31/55/30 33/65/74	25 0.005	50 0.01	LC-FLD	0.24/0.41/0.27 0.40/0.36/0.65	2019 [58]
China	AFB1 AFB2 AFG1 AFG2 AFM1 STER CIT FB1 FB2 OTA	Plasma	60 (30/30)C/HCC	13/33 17/23 3/3 3/3 3/0 13/40 0/3 3/7 3/3 0/3	0.07 0.05 0.13 0.15 0.16 0.05 0.18 0.41 0.39 0.15	0.25 0.21 0.43 0.38 0.41 0.22 0.44 0.92 0.87 0.46	LC-MS/MS	[0.95–1.78]/[1.23–4.56] [1.37–3.89]/[1.16–3.75] 0.61/0.55 0.43/0.46 0.57/n.d. [0.88–2.05]/[1.06–3.23] n.d./0.63 1.92/[1.35–2.78 ]2.03/1.57 n.d./0.83	2018 [64]
Italy	AFB1 AFM1 DON DOM-1 FB1 GLIO OTA ZEA	Serum	213	22.9 50.2 19.5 13.1 13.7 21.2 82.9 5.4	0.005 0.11 2.5 2.5 1.5 5.5 0.08 0.5	0.01 0.22 5.0 5.0 3.011 0.16 1.0	LC-MS/MS	0.01 [0–0.73] 0.11 [0–1.91] 1.0 [0–27.9] 0.3 [0–12.7] 0.7 [0–5.6] 2.3 [0–28.4] 0.36 [0–1.76] 0.1 [0–3.9]	2017 [82]
Portugal	OTA2’R-OTAEnB	Serum	42	100 81 100	0.012 0.012 0.0012	0.05 0.05 0.01	LC-MS/MS	0.76 [0.36–4.99] 0.32 [0.08–0.51] 0.048 [0.01–0.15]	2018 [60]
Germany	OTAEnB	Blood	50	100 100	0.012 0.0012	0.05 0.01	LC-MS/MS	0.204 0.036 [0.014–0.11]	2017 [59]
Spain	Ens (A,A1,B,B1) BEA	Plasma	10	0 0	0.01-0.04 0.02	0.02-0.04 0.04	LC-MS/MS	n.d. n.d.	2015 [111]
Germany	OTA	Blood	16	100	n.i.	n.i.	LC-MS/MS	0.157 [0.079–0.262]	2019 [63]
Czech Republic	OTA	Serum	50	48	0.04	0.10	LC-FLD	0.14 [LOD–0.83]	2019 [54]
Bangladesh	OTA OTα	Plasma	104	10098	0.050.05	0.100.10	LC-FLD	0.72 [LOD–6.63]0.38 [LOD–0.99]	2018 [55]
Egypt	OTA	Serum	98	81.6	0.2	n.i.	LC-FLD	0.33 [0.20–1.53]	2016 [85]
Italy	OTA	Serum	105 (62 C/43 CLD)	54.8/44.2	0.25	0.50	LC-FLD	0.26/0.27	2016 [118]
Germany	OTA	Blood	50	100	0.006	0.021	LC-MS/MS	0.211 [0.071–0.383]	2016 [61]
Germany	OTA 2’R-OTA	Blood	50 34	100 100	0.005 0.005	0.021 0.021	LC-MS/MS	0.21 [0.071–0.383] 0.11 [0.021–0.414]	2015 [62]
Bangladesh	CIT DH-CIT	Plasma	2	100 100	0.07 0.15	0.15 0.30	LC-MS/MS	0.47 [0.15–0.66] 0.96 [0.14–1.41]	2019 [56]
Czech Republic	CIT	Plasma	50	98	0.02	0.15	LC-MS/MS	0.05 [0.02–0.18]	2019 [54]
Bangladesh	CIT DH-CIT	Plasma	104	90 85	0.07 0.15	0.15 0.30	LC-FLD	0.34 [LOD–2.70] 0.38 [LOD–1.44]	2018 [55]
Italy	ZEA α-ZEL β-ZEL	Serum	110 (52 ASD/31 CS /27C)	n.d. n.d. n.d.	2.5 2.5 2.5	5 5 5	LC-MS/MS	<LOQ <LOQ <LOQ	2019 [58]
USA	ZEA α-ZEL β-ZEL ZAL ZAN	Serum	48	(free/conjugate) 85.4/100 6.3/62.5 35.4/39.6 16.7/75.03 1.3/93.8	0.07 0.07 0.07 0.07 0.07	n.i. n.i. n.i. n.i. n.i. n.i.	LC-MS/MS	(free/conjugate) 0.087/0.641 n.d./0.444 0.089/0.231 0/0.30 0102/0.203	2018 [68]
USA	ZEA	Serum	11	9	0.4 nM	n.i.	LC-MS/MS	0.39 nM	2016 [69]
Italy	AFs total (B1,B2,G1, G2, M1)	Serum	46	0	0.006-0.025	n.i.	LC-FLD	n.d.	2017 [86]
Turkey	AFs (B1,B2,G1,G2)	Serum	C: 49 CHB: 38 Cirr: 26 HCC: 35	26.5 21.1 26.9 35.0	0.025-0.05	0.021-0.06	LC-FLD	[0.005–0.018] [0.009–0.054] [0.010–0.041] [0.009–0.054]	2015 [57]
China	AFB1-lys	Plasma	260	19.6	0.5	1	LC-MS/MS	31.2 [10.5–74.5] *	2019 [65]
Mexico	AFB1-lys	Serum	34	83	0.35	0.47	LC-FLDLC-MS/MS	2.08 [1.08–102.6] *	2019 [81]
Bangladesh	AFB1-lys	Plasma	167	62	0.5 *	n.i.	LC-MS/MS	1.07 [0.04–123.5] *	2019 [115]
Uganda	AFB1-lys	Serum	220	100	0.2 *	n.i.	LC-FLD	5.83 [0.71–95.6] *	2019 [90]
Gambia	AFB1-lys	Plasma	374	95	3^E^	n.i.	ELISA	n.i.	2018 [79]
Malawi	AFB1-lys	Serum	230	67	2.5^E^	n.i.	ELISA	20.5 *	2018 [77]
Tanzania	AFB1-lys	Plasma	60	72	0.4 *	n.i.	LC-MS/MS	5.1 [3.5–6.6] *	2018 [113]
Mexico	AFB1-lys	Serum	347	99.4	0.2 *	n.i.	LC-FLD	0.82 *	2018 [91]
Nigeria	AFB1-lys	Plasma	58 (11 C/47 SAM)	19/81	0.022	0.022	LC-Orbitrap	0.8/4.3 [0.2–59.2] *	2018 [49]
Nepal	AFB1-lys	Plasma	85	n.i.	0.4 *	n.i.	LC-MS/MS	3.62 *	2017 [114]
Guatemala	AFB1-lys	Serum	461	100	n.i.	0.2 *	LC-MS	8.4 [0.2–814.8] *	2017 [112]
China	AFB1-lys	Plasma	459 (250 C/209 GBC)	15/32	0.5 *	n.i.	LC-MS	1.2/5.4 *	2017 [87]
Egypt	AFB1-lys	Serum	290	n.i.	n.i	n.i.	ELISA	[0.04-0.10] *	2016 [76]
Guinea	AFB1-lys	Serum	305	88.2	3^E^	n.i.	ELISA	12.1 *	2016 [78]
Malaysia	AFB1-lys	Serum	160	61	0.05	n.i.	LC-FLD	6.80 [0.80–20.24] *	2016 [89]
Uganda	AFB1-lys	Serum	713	90	0.4 *	n.i.	LC-FLD	1.58 [0.40–168] *	2015 [88]
Kenya	AFB1-lys	Serum	884	100	0.2 *	n.i.	LC-FLD	7.47 [6.04–8.90] *	2015 [92]
Gambia	AFB1-alb	Plasma	115	100	0.6^E^	n.i.	ELISA	3.6 [3.9–458.4] *	2015[75]
Tanzania	AFB1-lys	Plasma	166	67-98	3^E^	n.i.	ELISA	[4.7–23.5] *	2015 [117]

^a^ average concentration; ^E^LOD: supported by ELISA kit; *: pg/mg albumin; n.d: not detected; n.i: not indicated; 10-OH-OTA: 10-hydroxyochratoxin A; 2’R-OTA: 2’R-ochratoxin A; AFB1: aflatoxin B1; AFB1-alb: adduct of AFB1 with albumin; AFB1-lys: adduct of AFB1 with lysine; AFB2: aflatoxin B2; AFG1: aflatoxin G1; AFG2: aflatoxin G2; AFM1: aflatoxin M1; AFs: aflatoxins; ASD: autism spectrum disorder; BEA: beauvericin; C: control; CHB: chronic hepatitis B; Cirr: cirrhosis patients; CIT: citrinin; CLD: chronic liver disease; CRC: colorectal cancer; CS: control sibling; DH-CIT: dihydrocitrinone; DOM-1: deepoxy-deoxynivalenol; DON: deoxynivalenol; EnA: enniatin A; EnA1: enniatin A1; EnB: enniatin B; EnB1: enniatin B1; Ens: enniatins; FB1: fumonisin B1; FB2: fumonisin B2; GBC: gallbladder cancer; GLIO: gliotoxin; HCC: hepatocellular carcinoma; ID-MS: isotope dilution mass spectrometry; LOD: limit of detection; LOQ: limit of quantification; OTα: ochratoxin α; OTA: ochratoxin A; PAT: patulin; STER: sterigmatocystin; ZAL: zearalanol; ZAN: zearalanone; ZEA: zearalenone; ZEL: zearalenol.

**Table 4:** Most recent International Agency for Research on Cancer (IARC) classification for carcinogenicity and risk assessments carried out by different international agencies for each (group of) mycotoxin.

Mycotoxin	IARC Classification *	TDI Value
AFs	Group 1	Not established (genotoxic, carcinogen) [149]
FBs	Group 2B	1 µg/kg bw per day [150]
OTA	Group 2B	TWI: 100 ng/kg.bw per week [136] corresponds to 14 ng/kg.bw per day *Note: on- going (draft) scientific opinion, considered not appropriate to establish a TDI and that MOE approach needs to be applied (genotoxic, carcinogen)*[140]
STER	Group 2B	Not established (genotoxic, carcinogen) [151]
ZEA	Group 3	0.25 µg/kg.bw [152]
DON (including ADON and DON-glucoside)	Group 3	1 µg/kg.bw [153]
NIV	Group 3	1.2 µg/kg.bw ARfD: 14 µg/kg.bw [154]
T-2/HT-2	Group 3	0.02 µg/kg.bw ARfD: 0.3 µg/kg.bw [155]
CIT	Group 3	Level of no concern: 0.2 µg/kg.bw (large uncertainties, genotoxicity and carcinogenicity not excluded) [156]
PAT	Group 3	0.017 µg/kg.bw [157]
Ens and BEA	Not evaluated	Insufficient data to establish TDI or ARfD [146]

ARfD: acute reference dose; bw: body weight; TDI: tolerable daily intake; TWI: tolerable weekly intake; MOE: margin of exposure; ADON: acetyldeoxynivalenol; AFs: aflatoxins; BEA: beauvericin; CIT: citrinin; DON: deoxynivalenol; Ens: enniatins; FBs: fumonisins; HT-2: HT-2 toxin; NIV: nivalenol; OTA: ochratoxin A; PAT: patulin; STER: sterigmatocystin; T-2: T-2 toxin; ZEA: zearalenone. *IARC classification: AFs [158]; FBs [19,159]; OTA, ZEA, DON, NIV and T-2/HT-2 [19]; STER, CIT and PAT [160].

## Abbreviations

3-ADON	3-acetyldeoxynivalenol
15-ADON	15-acetyldeoxynivalenol
10-OH-OTA	10-hydroxyochratoxin A
2’R-OTA	2’R-ochratoxin A
ACN	acetonitrile
ADON	acetyldeoxynivalenol
AF-adducts	adducts of aflatoxins
AFB1	aflatoxin B1
AFB1-alb	adduct of AFB1 with albumin
AFB1-lys	adduct of AFB1 with lysine
AFB2	aflatoxin B2
AFG1	aflatoxin G1
AFG2	aflatoxin G2
AFM1	aflatoxin M1
AFM2	aflatoxin M2
AFOH	aflatoxicol
AFs	aflatoxins
ALT	altenuene
AME	alternariol monomethyl ether:
AOH	alternariol
APCI	atmospheric pressure chemical ionization
ARfD	acute reference dose
ASD	autism spectrum disorder
BEA	beauvericin
bw	body weight
CHB	chronic hepatitis B
CLB	chronic liver disease
CIT	citrinin
CRC	colorectal cancer
DAS	diacetoxyscirpenol
DH-CIT	dihydrocitrinone
DOM-1	deepoxy-deoxynivalenol
DON	deoxynivalenol
DON-3-GlcA	deoxynivalenol-3-glucuronide
DON-15-GlcA	deoxynivalenol-15-glucuronide
EDI	estimated daily intake
EFSA	European Food Safety Authority
ELISA	Enzyme-Linked Immunosorbent Assay
EnA	enniatin A
EnA1	enniatin A1
EnB	enniatin B
EnB1	enniatin B1
Ens	enniatins
ESI	electrospray ionization
EtOAc	ethylacetate
FB1	fumonisin B1
FB2	fumonisin B2
FBs	fumonisins
FDA	Food and Drug Administration
FLD	fluorescence detector
FUS-X	fusarenon-X
GBC	gallbladder cancer
GLIO	gliotoxin
HBGV	health-based guidance values
HBM	human biological monitoring
HCC	hepatocellular carcinoma
HRMS	high-resolution mass spectrometry
HT-2	HT-2 toxin
HT-2-4-GlcA	HT-2-toxin-4-glucuronide
IAC	immunoaffinity columns
IARC	International Agency for Research on Cancer
ID-MS	isotope dilution mass spectrometry
JECFA	Joint FAO/WHO Expert Committee on Food Additives
LC	liquid chromatography
LC-FLD	LC coupled with fluorescence detector
LC-HRMS	LC coupled with High-resolution mass spectrometry
LC-MS/MS	LC coupled with tandem mass spectrometry
LLE	liquid-liquid extraction
LOD	limit of detection
LOQ	limit of quantification
MeOH	methanol
MOE	margin of exposure
MRM	multiple reaction monitoring
MS	mass spectrometer
NIV	nivalenol
OTα	ochratoxin α
OTA	ochratoxin A
PAT	patulin
PBS	phosphate buffer solution
QqQ	triple quadrupole
QTrap	quadrupole-ion trap
QuEChERS	Quick Easy Cheap Effective Rugged and Safe
SRM	selective reaction monitoring
SAM	severe acute malnutrition
SPE	solid-phase extraction
STER	sterigmatocystin
T-2	T-2 toxin
TDI	tolerable daily intake
TWI	tolerable weekly intake
UHPLC	ultra-LC
ZAL	zearalanol
ZAN	zearalanone
ZAN-14-GlcA	zearalanone-14- glucuronide
ZEA	zearalenone
ZEA-14-GlcA	zearalenone-14- glucuronide
ZEL	zearalenol
